# TRAFFICGEN: a multi-agent LLM orchestration for smart mobility and emergency corridor pre-emption

**DOI:** 10.3389/frai.2026.1894645

**Published:** 2026-09-17

**Authors:** Gaurav Soni, Ganesh Khekare, Yash Kumar, S. Rupaak

**Affiliations:** School of Computer Science and Engineering, Vellore Institute of Technology, Vellore, Tamil Nadu, India

**Keywords:** agentic workflows, explainable AI, LangGraph, large language models, smart mobility, traffic signal control

## Abstract

This research work introduces TrafficGen, a proof-of-concept software prototype that explores the possibility of adaptive traffic signal management using LLM-based agentic orchestration. The framework is evaluated in a single-intersection, deterministic simulation environment that uses text to represent the various intersections and can operate with Gemini 2.5 Flash and LangGraph to implement a “Council of Agents” control architecture. TrafficGen uses a semantic translation layer to generate human-readable chain-of-thought reasoning logs for each signal decision, enabling it to manage complex traffic patterns. In empirical stochastic evaluations, TrafficGen was tested using four custom stress scenarios and 30 independent runs, yielding a mean reduction of 46.9% ± 2.8% in Wait Burden Score across all scenarios and runs (from 6,370 points to 3,380 points per run, compared to standard fixed-time signal controllers). Additionally, 100% of the Green Corridor maintained a success rate for dynamic emergency vehicle pre-emption. Architecturally, the system achieved a 100% decision override latency of 1,556 ms and a Green Corridor success rate of 100% for life safety evaluations. The quantitative results show the effectiveness of zero-shot semantic LLM orchestration compared to other LLM controllers with heuristic rules. These results come from a controlled single intersection environment and may not guarantee similar performance when deploying traffic management in municipalities generally, but TrafficGen paves the way for traffic management to move beyond black-box numerical paradigms and into explainable decision-making. Finally, this paper is an initial step towards adaptive traffic management for smart urban mobility.

## 1 Introduction

### 1.1 Background

The rapid pace of urbanization worldwide has transformed modern transportation, stretching current infrastructure to its physical and operational limits. By 2026, the concept of the Smart City has evolved from a theoretical model into a data-rich reality, with billions of IoT sensors, connected vehicles (CVs), and smart infrastructure generating a torrent of high-velocity data. The critical question is: how can we turn this big data stream into dynamic, secure, and effective mobility?

Traditionally, traffic control relied on past trends and localized control. We are now entering an era where generative AI and multi-agent systems (MAS) can generate and anticipate complex human behavior even before it manifests on the road. TrafficGen represents this technological transformation, shifting towards an active, self-negotiating ecosystem rather than passive, reactive stop-and-go logic. By applying the machine learning agent concept to each object, such as a delivery drone or a commuter car, we can transition to more complex automation and achieve genuine urban orchestration.

#### 1.1.1 Introduction to the problem domain

Urban mobility is characterized by non-linearity and stochasticity. In a metropolitan gridlock, a single event, such as a double-parked delivery truck or a heavy rainstorm, can trigger a butterfly effect that spreads across the entire city and causes gridlock. This domain is marked by several critical bottlenecks, such as

- Scalability Paradox: Traditional traffic models achieve good results at isolated intersections but do not scale to a network of hundreds of interacting nodes. The computational complexity of coordinating agents (vehicles and signals) increases exponentially with the number of agents.
- Behavioral Unpredictability: Traffic is not just a stream of metal, but also a stream of human choices. Pedestrians may jaywalk, and drivers can make unpredictable maneuvers. Classical mathematical models can hardly capture these long-tail edge cases.
- Environmental and Economic Leaks: Congestion is not only a nuisance but also a leading contributor to carbon emissions in urban areas. Poorly timed signals lead to excessive idling, while inefficient routing results in high fuel consumption. Economically, billions of dollars are wasted each year in productivity due to time lost during transit.
- Rigidity of infrastructure: Physical infrastructure (roads, bridges) is fixed and costly to modify. Therefore, the” intelligence” should be concentrated in the software layer—the management system—to optimize the utility of existing physical assets through adaptive logic.

The domain needs a system that does not simply observe the current state of traffic but can envision possible future states and optimize over those in real time.

#### 1.1.2 Current systems and existing solutions

Existing traffic management systems can be broadly grouped into three categories, each with its limitations:

1. Rule-Based Signal Control (e.g., SCATS, SCOOT): These industry standards utilize inductive loop sensors to detect the presence of vehicles and adjust signal timing based on set thresholds. They are reliable but reactive, unable to anticipate an influx of traffic until it occurs, and lack the capability to make complex trade-offs between different modes of transport (e.g., between a bus and five cars).
2. Microsimulation Tools: Urban planners use these tools to simulate traffic flows. They operate as closed-loop systems; they can predict potential outcomes using past data but struggle to incorporate real-time behavior over time.
3. Early-Stage AI and Single-Agent RL: In recent years, reinforcement learning (RL) has been introduced to traffic management. Nevertheless, most implementations are single-agent systems where a central AI attempts to manage an entire city. This creates a single point of failure and requires substantial centralized computing resources. Moreover, these models often lack a generative component, meaning they can only maximize scenarios they have encountered in their training data and are fragile when faced with unique real-world anomalies.

TrafficGen addresses these shortcomings by decentralizing intelligence (multi-agent) and providing the ability to synthesize unseen scenarios (generative AI).

In addition, for the research work, we have the following:

- Generation Evolution: For centuries, traffic control has progressed through three different generations. The first-generation systems used fixed-time, pre-programmed signal timing plans based on historical traffic volumes—without adaptivity. Actuated, sensor-based controllers (e.g., SCATS and SCOOT) were introduced in Generation 2 to allow the green phase time to be adjusted in real time according to information from loop detectors and/or vision sensors. Recently, Generation 3 systems have been based on deep reinforcement learning (DRL) and multi-agent reinforcement learning (MARL) to continually learn signal timing policies using deep neural networks in high-dimensional state spaces.
- The Semantic Void: There is a fundamental problem with traditional MARL architectures, termed the semantic void. This critical issue arises because standard MARL algorithms cannot understand the physical environment, as traffic is observed merely as a scalar matrix of numbers. Consequently, a Generation 3 model cannot semantically distinguish a standard high-density commuter queue from a critical vehicle blockage in an emergency situation when both have the same scalar density value. This abstraction gap makes it challenging for traditional MARL systems to consider domain-specific context or to explain their reasoning in a human-readable way, particularly concerning safety-critical signal actions.

The general background and related context on smart mobility are provided by standard deep reinforcement learning (DRL) architectures, as well as generative diffusion models. TrafficGen examines LLM-driven semantic agent orchestration as an alternative smart mobility control paradigm. The proposed framework is based on zero-shot semantic reasoning and natural language state representations, enabling explainable and real-time traffic signal optimization and dynamic life-safety pre-emption, rather than relying on black-box numerical policy maps or complex continuous state-space optimizations.

### 1.2 Motivation

main driving force behind trafficking is the necessity to bridge the gap between fixed city planning and the reality of mobile mobility. Traffic management in cities is approaching zero margins as urban density increases. The potential of Generative AI as a crystal ball, enabling the simulation of millions of what-if scenarios in a digital twin environment, and only altering a single signal in the physical world, motivates this model.

Additionally, the transition to autonomous and connected cars requires a system capable of communicating in their language. TrafficGen aims to develop a so-called Universal Traffic Language through which infrastructure and vehicles negotiate space and time. This collaborative effort is essential for transitioning from egoistic driving (where each car attempts to save time) to socially optimal driving (where the entire network travels faster).

The environmental impact is negligible. We can significantly reduce the carbon footprint of each trip by minimizing the stop-and-go nature of urban driving through adaptive signal control and smart routing.

Moreover, we are driven by safety. A high proportion of accidents in urban areas occur at intersections due to conflicting intentions. Utilizing pedestrians as agents in the generative model allows it to anticipate perilous interactions in advance, modifying signal timings or notifying connected vehicles to decelerate ahead of time.

Although urban signal optimization directly impacts vehicle emissions, the environmental benefits in this study—including lower fuel consumption and reduced carbon footprint from decreased vehicle idling durations (Wait Burden Score reductions)—are modeled as derived theoretical estimates rather than directly measured tailpipe emissions.

Finally, this research is fueled by the democratization of smart mobility. TrafficGen can be implemented in new Smart Cities where decades of historical data required by traditional systems may not be available, by generating models that can be trained with minimal real-world data to extrapolate the rest.

### 1.3 Scope of the research work

Considering the limitations of the system and the scope of the experiments, this research is clearly divided into two categories:

#### 1.3.1 Implemented & validated scope

The key empirical work applied and tested in the context of this study includes

- LLM-Based State-Graph Orchestration: Multi-agent decision logic orchestrated with LangGraph and powered by the main cognitive engine, Gemini 2.5 Flash.
- Semantic Translation Layer: Conversion of discrete traffic state parameters into structured prompt context and chain-of-thought (CoT) logs for natural language reasoning.
- Single-Intersection Digital Twin: A text-based, deterministic simulation environment to test the adaptive signal control system under extreme lane starvation.
- Emergency Vehicle Pre-emption: Dynamic priority signal override for life-safety corridors. Explainable AI (XAI) Auditing: Creation of a log of AI reasoning that is human-readable and auditable for each signal phase decision.
- Practical Infrastructure Interface: Provides an actionable deployment roadmap that enables high-level LLM reasoning to be integrated into physical civil infrastructure through a low-bandwidth summary of perception at the edge, converted into standardized API calls for controlling the infrastructure (e.g., set_light_green) without necessitating total hardware changes.

#### 1.3.2 Future & extended architectural vision

The broader long-term system architecture envisions a more comprehensive capability that extends beyond the single intersection Proof of Concept, outlining directions for future enhancement:

- Generative diffusion models for visual scene synthesis and edge sensor data simulation: latent diffusion scene synthesis.
- Multi-intersection MARL coordination: distributed MARL synchronization and semantic orchestration in multi-node traffic networks.
- Multimodal pedestrian density monitoring and crosswalk timing via pedestrian-level micro-tracking using computer vision feeds.
- Edge deployment: Model quantization (4-bit/8-bit) and optimization for local execution to reduce API cloud latency on edge devices such as NVIDIA Jetson.

## 2 Research work description and goals

### 2.1 Literature review

From basic mechanical timers to highly adaptive systems powered by artificial intelligence, the evolution of traffic signal control (TSC) systems illustrates an ongoing quest to improve urban mobility. This literature review summarizes this trend and identifies the main challenges encountered in the existing SOTA reinforcement learning methods, while also laying the groundwork for the use of generative AI and large language models (LLMs) in cyber-physical infrastructure.

Early work in intersection management primarily focused on the fixed-time deterministic approach, often implemented through Webster’s formula for determining cycle lengths to minimize delay (Webster, 1958). Although these methods were essential, they were inflexible and unable to cope with the stochastic variations in traffic volume. Physical induction loops were deployed, and manuals were developed for traffic engineering to standardize actuated control (Roess et al., 2004; Koonce et al., 2008). In the late 1980s, adaptive traffic control systems (ATCS) were designed to address the non-stationary traffic of urban networks, such as the Split Cycle Offset Optimization Technique (SCOOT) (Hunt et al., 1981) and SCATS (Sydney Coordinated Adaptive Traffic System) (Lowrie, 1990). These systems use heuristic algorithms to dynamically change signal phases based on actual sensor data. However, they cannot respond to micro-level, zero-shot anomalies like sudden multi-vehicle incidents.

The introduction of deep learning has led to a paradigm shift from traditional model-based methods to reinforcement learning (RL) for traffic optimization in control systems (El-Tantawy et al., 2013). RL agents learn optimal policies by continuously interacting with simulated environments based on Markov decision processes (Sutton and Barto, 2018). As the size and complexity of intersection networks increased, the use of isolated RL was found insufficient, leading to the introduction of multi-agent reinforcement learning (MARL) (Chu et al., 2020).

In recent years, MARL has emerged as the SOTA for traffic signal control, as evidenced in the literature. Wei et al. presented PressLight, which optimized signals by computing the “max-pressure” between incoming and outgoing lanes (Wei et al., 2019a). Subsequently, CoLight used graph attention networks for spatial communication between adjacent intersection agents, achieving significant improvements in network-level flow (Wei et al., 2019b). Meta-Learning, developed by MetaLight, addresses the generalization problem across numerous intersection topologies within MARL frameworks, enabling agents to learn quickly for new traffic distributions (Zang et al., 2020). Other notable frameworks, such as FRAP (Zheng et al., 2019), utilized phase competition modeling to enhance agent learning.

While MARL systems may perform well in simulation, they face significant deployment challenges (Dafoe et al., 2021). They suffer from the “curse of dimensionality”, necessitating vast numbers of compute nodes (GPU clusters) to train in continuous state spaces (Chu et al., 2020). Most importantly for public infrastructure, MARL networks are “black boxes” (Guidotti et al., 2018). If an RL agent makes an erroneous action or decision due to an accident, it becomes challenging for human auditors to comprehend the rationale behind the decision based on the underlying neural weights (Barredo Arrieta et al., 2020).

The emergent capabilities of natural language processing (NLP) models, typically trained to generate text, have shown that large language models (LLMs) can possess emergent reasoning and logic abilities (Brown et al., 2020; Bubeck et al., 2023) to overcome the constraints of RL. LLMs can break down complex, multi-step optimization problems into logical, sequential deductions through various methods such as chain-of-thought (CoT) prompting (Wei et al., 2022).

LLMs can now interact with the outside world, reasoning and acting as represented in the reasoning and acting (ReAct) paradigm (Yao et al., 2023). This ability has been advanced by models, including Google’s Gemini, which deliver ultra-low latency and multimodal inference essential for real-time applications (Gemini Team et al., 2023). LLMs are currently being studied for use in autonomous driving systems and transportation routing (Xu et al., 2024), where semantic reasoning shows great promise for understanding complex spatial rules without specialized mathematical training.

Recently, the concept of single LLMs has been extended into multi-agent systems (MAS), where multiple personality-specific LLMs collaborate to achieve complex tasks (Wang et al., 2024). For instance, AutoGen and LangGraph enable decentralized negotiation, with agents exchanging contextual states, discussing results, and reaching a consensus based on algorithms (Wu et al., 2023; Dafoe et al., 2021). This multi-agent semantic architecture can be directly applied to urban traffic, with specialized agents (standard optimizers vs. emergency pre-emptors) performing functionally on the right-of-way using deterministic logic (without a probabilistic reward) (Han et al., 2016).

Explainable AI (XAI) is a significant benefit of LLM-powered control systems. In the RL domain, explainability is a complex post-hoc surrogate model (Lin et al., 2024), while for LLL, explainability is built into the LLM’s capability to produce natural language logs that describe its cognitive reasoning before taking action, which is necessary for civil infrastructure auditing. Additionally, the literature suggests that Edge-AI deployment addresses the latency and connection concerns of cloud-based APIs. Recent studies have demonstrated that powerful LLMs can now run locally on low-resource edge devices, delivering deterministic, low-latency performance in decentralized settings, using activation-aware weight quantization (AWQ) and four-bit model compression (Khetan et al., 2025).

The proposed TrafficGen system fills the gaps in the literature by replacing the opaque and heavy computation of MARL models with a lightweight, transparent, and linguistically reasoned “Council of Agents”.

The general purpose of the updated comparison in Table 1 is to move beyond the misguided quantitative scalar comparison of traffic signal control (TSC) paradigms and towards a comprehensive conceptual and architectural comparison (Khekare et al., 2025b). Traditional multi-agent reinforcement learning (MARL) benchmarks (PressLight, CoLight, and MetaLight) are based on fixed state representations and optimization of execution policies using continuous numerical reward functions such as max-pressure gradients, graph attention networks, or gradient-based task adaptation. While these traditional MARL methods excel at numerical computation in limited training contexts, their internal decision-making logic is difficult to interpret from outside the MARL and is tightly wrapped in the weights of deep neural networks, which are easily affected by distribution shifts (Khekare, 2014).

**Table 1:** Conceptual comparison of traffic signal control (TSC) frameworks.

Paradigm/framework	Control foundation	Adaptability mechanism	Operational interpretability	Primary operational trade-off
PressLight	Multi-agent RL (Max-Pressure)	Mathematical state dynamics	Opaque (neural weights)	Very efficient at mathematical performance; zero-shot distribution shift sensitivity
CoLight	Multi-agent RL (graph attention)	Spatial graph communication	Opaque (neural weights)	Good spatial modeling; poor in non-graph topological changes
MetaLight	Multi-agent Meta-RL	Gradient-based task adaptation	Opaque (neural weights)	Provides quick multi-task transfer but has risk of reward function misalignment
TrafficGen (proposed)	GenAI-agentic framework	Zero-shot semantic reasoning	Transparent (natural language XAI logs)	Depends on inference latency management and has high qualitative contextual reasoning

In contrast, TrafficGen proposes a paradigm shift in the underlying control system by using Generative AI-based agents. TrafficGen uses high-level zero-shot semantic reasoning to determine actuation based on messages it translates from raw traffic telemetry to natural language state serializations (Khekare et al., 2025a). This approach alters the trade-off landscape: while classical MARL can prioritize explainability and mathematical efficiency while maintaining short inference execution times, TrafficGen emphasizes explainable, human-auditable decision logs (via chain-of-thought reasoning) and strong long-tail adaptivity at the expense of language model inference time. The table highlights that these two paradigms differ in fundamental aspects of control, namely control foundation, adaptability mechanism, operational interpretability, and primary operational trade-offs. TrafficGen is distinct in its approach but does not provide a point-by-point numeric comparison of different non-identical control abstractions.

### 2.2 Research gap

However, even with the rapid development of AI-driven traffic management, several critical gaps remain that prevent the implementation of a properly intelligent, self-driven urban mobility ecosystem.

1. What-If Simulation Bottleneck. Most existing multi-agent reinforcement learning (MARL) systems are trained on past data or on simulators, such as SUMO, which are not dynamic. These systems are responsive rather than generative. Traditional models fail when real-world anomalies occur (e.g., a massive road closure because of a sudden sinkhole) since they have never encountered such states. There is no unified system that applies generative models for synthesizing extreme edge cases in real time to pre-train or fine-tune decision-making agents before a crisis arises.
2. Failure to Pay Attention to Heterogeneous Agent Interaction. Conventional traffic models tend to focus on vehicles as the primary actors, relegating pedestrians and cyclists to background noise (or immobile objects). However, in a smart city, people on the street are the negotiators. Existing literature lacks a single MARL system where pedestrian agents and vehicle agents are trained to cooperate and learn to negotiate (e.g., a car moving forward to signal intent for a pedestrian to wait, or a signal agent prioritizing a high-density pedestrian crossing to reduce total human wait time).
3. Real-Time Latency vs. Model Complexity: Diffusion models (or large transformer models) are computationally costly. Their inference time can exceed the required 100 *ms* for real-time traffic signal control, but they generate high-fidelity scenarios. Research on how to distill generative knowledge into lightweight, edge-compatible agents capable of acting at intersections without significant cloud-computing overheads has a significant gap.
4. Absence of “Proactive” Digital Twin Synchronization. Although digital twins (DT) are a reality, they are often applied in monitoring but not in proactive intervention. The majority of systems align the virtual state with the physical state but do not execute “congestion-predictive” generative forward passes to predict congestion 15 min in advance and modify the current signal phases accordingly. The predictive-only gap remains an obstacle to preventing gridlock before it occurs.

### 2.3 Objectives

The main objective of the TrafficGen research work is to develop a powerful, generative, and collaborative multi-agent system that can maximize urban mobility. Its objectives are

1. Generate a Generative Traffic Scene Engine: To create an autoregressive generative model that can produce 10,000 + varied traffic scenes, including edge cases such as accidents and extreme weather, to be used as a training environment for RL agents.
2. Introduce a Decentralized MARL System: To develop a “Centralized Training, Decentralized Implementation” (CTDE) system in which traffic lights and self-driving cars can be viewed as autonomous entities that can negotiate locally optimal flows and comply with international efficiency standards.
3. Include Pedestrian-Vehicle Cooperation: To make pedestrians the first-class agents of the simulation, it is essential to ensure that the AI maximizes safety and minimizes wait time for all forms of transport.
4. Real-Time Edge Efficiency: To fine-tune the model architecture with knowledge distillation and quantization, the inference latency should be less than 80 *ms* on typical edge-AI hardware (e.g., NVIDIA Jetson).

Furthermore, the other objectives are listed below:

- Design and validate a cognitive multi-agent system (MAS) transitioning control from static rules to semantic reasoning.
- Target a ≥ 20% reduction in average waiting time (AWT).

### 2.4 Problem statement

Urban centers are experiencing a Mobility Paradox: the volume of vehicles and pedestrians is growing exponentially, yet physical infrastructure (roads and intersections) is not. Conventional traffic management systems are reactive and rule-based; they can only respond to congestion after it has already occurred. These systems rely on timers with fixed cycles or simple loop detectors that do not differentiate between an ambulance, a bus, or a private car, creating significant inefficiencies.

Moreover, existing traffic simulators cannot replicate the long-tail distribution of human behavior. Uncommon but significant incidents, such as a pedestrian jaywalking on a highway or a car stalling in a tunnel, cause systemic failures in current AI models due to their inability to generate such events. This results in:

- Economic Loss: Billions of dollars in lost productivity because of traffic delays.
- Environmental Degradation: Overproduction of CO_2_ by stop-and-go traffic.
- Safety Hazards: A high incidence of accidents at intersections due to a lack of coordination between motorized and non-motorized agents.

TrafficGen aims to address this by transforming traffic management from a reactionary signal-timer problem into a proactive generative negotiation challenge.

### 2.5 Research work plan

The TrafficGen research work plan is a technical roadmap for shifting theoretical modeling to a hardware-verified deployment of a digital twin. Given the complexity of combining generative AI and multi-agent reinforcement learning (MARL), the plan is structured modularly, with each part stress-tested separately before integrating the entire system.

Phase 1: Foundation and Data Engineering. This phase focuses on creating a robust data backbone to capture the chaotic reality of 2026 urban mobility.

- Data Aggregation: Aggregate high-fidelity telemetry from open datasets and augment it with real-time vehicle-to-everything (V2X) logs from partner smart-city sensors.
- Behavioral Feature Extraction: Use unsupervised learning to classify driving styles (e.g., aggressive, cautious, distracted) and pedestrian patterns, ensuring that the generative model encompasses a wide range of behaviors. Spatio-Temporal Mapping: Transform unstructured GPS and LiDAR data into structured vector maps, determining the topology of intersections, lane connectivity, and pedestrian conflict zones.
- Milestone: The Unified Traffic Data Lake is completed and optimized for high-speed training.

Phase 2: Generative Scenario Synthesis. During this stage, we develop the system’s imagination—the generative core that enables the AI to model an infinite number of what-if situations.

- Model Architecture: Train a Latent Diffusion or Auto-Regressive Transformer on images of generated traffic scenes. This model will generate initial vehicle positions and intentions based on time of day and weather, unlike conventional simulators.
- Edge-Case Injection: Specifically train the model to produce long-tail cases, such as a vehicle stalling in a high-speed lane or a rush of people around a stadium event.
- Validation: Compare the generated traffic statistical distribution with historical data from the real world to validate plausibility using Fréchet Inception Distance metrics. Milestone: Deployment of the TrafficGen Scenario Engine API.

Phase 3: Intelligence and Negotiation. This is the core development stage, during which the agents learn to maneuver and control the created situations.

- Specialization of Agents: Signal Agents: Learn how to maximize “Green Waves” and reduce idling time. Vehicle Agents: Negotiate for safety and optimal routes based on decentralized negotiation. Pedestrian Agents: Prioritize safety and speed of crossing.
- Reward Shaping: Develop a multi-objective reward mechanism that balances throughput (travel time), sustainability (fuel consumption/CO_2_), and safety (collision proximity).
- Collaborative Training: Train multi-agent proximal policy optimization (MAPPO) in the generative context, where agents learn by interacting with scenarios generated by the model.
- Milestone: A library of pre-trained cooperative weights that can operate a typical 4×4 urban grid.

Phase 4: Digital Twin and Dashboard Phase. The emphasis shifts from training to live synchronization between the real world and the AI model.

- Real-Time Sync Engine: Build the pipeline that streams sensor data to the MARL agents in real time, enabling them to make real-time signal corrections.
- Predictive Intervention Layer: Add a Fast-Forward to Simulation option. In case of a detected anomaly, the system will execute 100 generative simulations 15 min into the future to determine the best intervention.
- Visual Interface: Develop a fast dashboard to display city operators with heatmaps of congestion, agent trust ratings, and estimated CO_2_ savings.
- Milestone: TrafficGen Digital Twin v1.0 operational in a virtual city environment.

Phase 5: Evaluation, Optimization, and Deployment. The final step is to prepare the system for real-world infrastructure.

- Model Quantization: Train the large generative and MARL models to operate on Edge-AI devices (e.g., NVIDIA Jetson Orin) at the traffic intersections themselves.
- Benchmarking: Conduct stringent tests against standard traffic control algorithms (SCATS/SCOOT). The aim is to effectively reduce average wait times and urban emissions.
- Safety Audit: Perform “Red Teaming,” in which the system is deliberately subjected to corrupted data to ensure it can safely recover to a standard fixed-time cycle.
- Milestone: Final system validation report and deployment-ready software package.

## 3 Technical specification

This section outlines the technical details of the traffic management system, generative AI, multi-agent system, and traffic forecasting. It addresses the technical specifications, architectural parameters, and feasibility constraints of the proposed generative AI (GAI)-based multi-agent traffic management system (TrafficGen). The system requirements reflect a new paradigm of semantic reasoning, explainable AI (XAI), and orchestration of the state-graph, contrasting with the previously discussed multi-agent reinforcement learning (MARL) paradigms, which were computationally intensive.

### 3.1 Requirements

The system requirements are divided into functional and non-functional categories. Functional requirements specify the deterministic behavior of the multi-agent environment, while non-functional requirements outline the necessary levels of performance, security, and scalability for deploying the system in an urban setting. This section defines the “What” and the “How well” of TrafficGen.

#### 3.1.1 Functional requirements

The functional requirements will outline the necessary operations the system must perform to convert raw intersection telemetry data into actual traffic light control.

- Multi-Modal Telemetry Ingestion & Semantic Translation: The system shall actively ingest data from various Layer 1 physical sensors (IoT cameras, inductive loops, LiDAR) and translate the data semantically. Most importantly, the Perception Module must dynamically convert a quantitative number array (queue length, wait time, etc.) into a structured semantic text prompt that can be interpreted by the LLM.
- Hierarchical Agent Orchestration: The system should utilize a directed (LangGraph) state-graph architecture with specialized agents. A Supervisor Agent is required to continuously assess the type of semantic context received and direct the execution flow to an IntersectionAgent for normal load balancing or to an EmergencyVehicleAgent for priority overrides.
- Dynamic Conflict Resolution & Starvation Prevention: The IntersectionAgent should function as a Dynamic Conflict Resolution and Starvation Prevention system, evaluating conflicting traffic demands. It must compute trade-offs between the number of vehicles in a queue and the maximum waiting time allowed to dynamically program the duration of the green light without any single lane experiencing “starvation.”
- Pre-emptive Emergency Override: The system should detect [EMERGENCY VEHICLE DETECTED] flags in the context vector. If detected, the EmergencyVehicleAgent should have the authority to immediately use the override_signal tool, which overrides the normal cycle to establish a Green Corridor.
- Explainable AI (XAI) Log Generation: A human-readable, natural language log must be produced for each actuation decision. The log should clearly indicate the context provided to the AI, how the AI reasoned through its process, and how the AI reached its final decision.
- Emergency Pre-emption Verification and False Alarm Mitigation: The framework shall include an automatic false alarm verification sub-routine to help reduce false positive pre-emption signals (e.g., when an emergency vehicle stops or changes lanes before reaching the intersection and turns off its siren). If an override request is received from the EmergencyVehicleAgent, the system shall display a user-configurable confirmation window (Time_verify = 15 s). If secondary verification (by camera computer vision feeds or V2X spatial telemetry) cannot confirm the actual vehicle arrival within Time_verify, the system shall promptly end the pre-emption, document the false alarm event in the explainable artificial intelligence (XAI) audit trail, and revert to standard signal control for the IntersectionAgent.

Table 2 presents a Functional Requirements Traceability Matrix, which relates the core capabilities of the TrafficGen system to their respective modules and prioritizes each capability. It assigns the EmergencyVehicleAgent’s managed life-safety functions the highest “Critical” priority. Their respective agents are marked as “High” priority for standard operational functionalities like telemetry translation, state-graph routing, starvation prevention, etc. Lastly, the ExplainableAILogger generates clear audit logs, which is classified as a “Medium” priority task in the system architecture.

**Table 2:** Functional requirements traceability matrix.

Description	Primary agent/module	Priority
Semantic telemetry translation	Perception module	High
State-graph routing	SupervisorAgent	High
Starvation prevention	IntersectionAgent	High
Emergency pre-emption	EmergencyVehicleAgent	Critical
XAI audit generation	ExplainableAILogger	Medium

#### 3.1.2 Non-functional requirements

The non-functional requirements outline the constraints and quality attributes to consider when designing the TrafficGen architecture, particularly in a mission-critical urban environment.

Ultra-Low Latency & Throughput: It must be possible to ingest telemetry, use the LLM, and then actuate the tool within acceptable human-reaction time limits. The prototype phase utilizes cloud-based APIs (e.g., Gemini 2.5 Flash) and will be followed by eventual 4-bit/8-bit edge quantization, which will reduce decision latency to less than 100 ms.

Deterministic Fallback (Reliability): The system should ensure that it is consistently up to date and available at 99.999% with a Rule-Based Round-Robin controller instead of the LLM whenever the API is rate-limited, fails, or hallucinates. A road traffic stop cannot be caused by software failure.

Scalability (Decentralized Negotiation): The architecture needs to be horizontally scalable. Eventually, agents at a particular intersection must be able to pass a small amount of token state information to adjacent intersections to facilitate “proactive green waves”.

Data Privacy & Compliance: The system should not collect raw visual data to comply with urban surveillance laws. Only anonymized telemetry (car count) should be retained, and the physical identifier should be removed as soon as possible within the context of federated learning.

Operational Latency and Response Metrics: To achieve system clarity and physical feasibility, the operational requirements clearly separate the operational latency of the LLM API from the edge latency of the target physical hardware:

- Macro-Level Cloud API Latency (Proof-of-Concept): The current prototype software for cloud support (PoC) API decision response latency ranges from 1,500 ms to 6,371 ms. This latency profile is designed for macro-level cycle adjustments, long-term phase scheduling for signal timing, and digital twin scenario forecasting with signal timing modifications in multi-second or multi-minute time windows, rather than in the microsecond control loop.
- Micro-Level Edge Actuation Latency (Physical Deployment Target): Cloud network transmission overhead is not feasible for micro-level edge actuation latency (physical deployment target) for real-time physical signal switching and microsecond safety loop actuation at physical intersections. For municipal deployment, the inference latency should be less than 100 ms, achieved through local edge processing instead of cloud infrastructure.

### 3.2 Feasibility study

A thorough feasibility study was conducted to assess the transition from MARL to an LLM-based Council of Agents. Technical, economic, and operational considerations were examined.

#### 3.2.1 Technical feasibility

Technically, the research is eminently solvable due to the latest developments in generative AI inference speed and state-graph frameworks. The “curse of dimensionality” in traditional multi-agent reinforcement learning (MARL) makes it challenging to simulate traffic control problems with continuous state space, such as those from MegaDrive simulations, without using large clusters of GPUs. We aimed to convert a continuous physics problem for the study into a discrete semantic logic puzzle by utilizing LLMs to replace the complex mathematical models in the research. The software architecture is highly feasible, supported by existing libraries, such as LangChain and StateGraph, that enable LLM reasoning to be bound to real hardware, with the usage tool being a Python execution program (set_light_green), thus avoiding the significant hardware dependency of Phase 1 MARL approaches.

#### 3.2.2 Economic feasibility

The economic viability of the system addresses the “Mobility Paradox” (the notion that increasing physical mobility is economically and geographically untenable).

- Deployment Costs: Traditional smart lights require expensive local computing. By using lightweight LLMs, cities can leverage existing induction loop and camera infrastructure, transmitting minimal text data payloads to cloud APIs (or inexpensive localized Edge TPUs), significantly reducing capital expenditure (CAPEX).
- The capstone benchmark indicated a 46.9% decrease in “Wait Burden.” The system helps alleviate urban congestion, which indirectly lowers millions of dollars in lost economic productivity, fuel usage, and CO_2_ emissions, making its return on investment (ROI) highly favorable for city councils.

#### 3.2.3 Operational feasibility

The “Black Box” problem is the greatest challenge to AI in public infrastructure; city planners cannot deploy algorithms that lack clarity on their decision-making processes in case of an accident. This system is highly operationally feasible, thanks to the use of explainable AI (XAI). The agents “think” in natural language, which allows them to automatically produce human-readable audit logs of all the light changes they make. This transparency meets legal audit conditions, fosters public trust, and enhances the viability of operational adoption.

The TrafficGen system demonstrates a high level of research viability, as indicated by the Feasibility Risk Assessment shown in Table 3, which covers three primary areas: Technical, Economic, and Operational. It identifies LLM hallucinations as the most significant technical threat, which is mitigated by hardcoded rule-based overrides and strict typing when using tools. API tokens pose a recurring cost challenge, addressed through a planned transition to Edge-AI quantization that avoids additional costs and enables local inference. Finally, the challenge of public and legal trust is addressed by implementing full logging in natural language XAI, which comprehensively tackles this issue. All assessed domains have received a “High” or “Very High” viability score due to these robust mitigation measures.

**Table 3:** Feasibility risk assessment.

Feasibility domain	Primary risk factor	Mitigation strategy	Viability score
Technical	LLM hallucinations	Hardcoded rule-based overrides & strict tool-use typing.	High
Economic	API token costs	Transition to Edge-AI quantization for local, free inference.	High
Operational	Public/legal trust	Implementation of comprehensive XAI natural-language logging.	Very high

### 3.3 System specification

The system specification is divided into five sub-sections, detailing the overall integration of the TrafficGen architecture blueprint.

#### 3.3.1 Layered architectural specification

It utilizes a modernized 4-layer architecture and deviates from the Latent Diffusion MARL pipeline:

- Layer 1 (Perception): Physical sensors provide sensory input, collecting V2X telemetry and IoT data, and sending it in a standardized JSON payload that conveys information on queue length, wait times, and emergency statuses per lane.
- Layer 2 (Generative Core): The Perception Module converts the Layer 1 JSON into semantic text in Layer 2 (Generative Core). This becomes the “Imagination” layer, which helps the AI understand the real world.
- Layer 3 (Decision Engine): LangGraph state machine. In this case, Gemini 2.5 Flash serves as the analytical component that evaluates the Layer 2 text with custom prompts to ensure optimal flow.
- Layer 4 (Application): The application layer where the AI’s intentions are converted back to the physical world using Python tools, updating the 3D digital twin and altering the physical light statuses.

#### 3.3.2 Data schema and state flow specification

The Data Schema and State Flow Specification are outlined below.

Data continuity is achieved by passing a single AgentState dictionary from LangGraph node to LangGraph node.

- Input Schema: {“intersection_id”: String, “lanes”: [{“lane”: String, “queue”: Int, “max_wait”: Int, “emergency”: Boolean}]}
- The state graph schema captures the LLM’s chain of thought, adds an xai_log string with the justification, and includes an action_intent variable containing the final Python function call as it flows through the AI.

#### 3.3.3 Multi-agent workflow specification

The decision-making process is based on a deterministic state machine rather than a black-box neural network.

- Intake: This is where all data is directed to the SupervisorAgent.
- Supervisor: The Supervisor classifies the data into either STANDARD_ROUTING or EMERGENCY_ROUTING.
- Standard: The IntersectionAgent is given the state and uses the optimization prompt to balance the North, South, East, and West queues, writing the duration to the set_light_green tool as a variable <duration>. In the case of an emergency, the EmergencyVehicleAgent bypasses optimization and searches for the longest wait time of all conflicting emergencies, outputting a < lane_id> for the override_signal tool.

#### 3.3.4 Hardware and edge-deployment specification

The prototype benchmark uses cloud-based API calls to Google Gemini, whereas the production deployment specification uses Edge-AI quantization.

- Local neural processing units (NPUs) or Edge TPUs should be installed in intersection control boxes.
- All LLM models will be quantized to 4-bit precision, enabling full processing of the multi-agent state graph locally, without the need for an internet connection. This hardware specification guarantees a response time of less than 100 milliseconds and helps avoid traffic congestion during urban internet outages. Additionally, upcoming versions will have direct integration of vision-language models (VLMs) into edge cameras for multi-modal perception, without the need for telemetry translation.

#### 3.3.5 Security, trust, and fail-safe specification

The system specification provides a hardened fail-safe mechanism due to the life safety concerns of traffic control.

- Safety Envelope: A hard-coded boundary limit exists in the Python action tools set_light_green and override_signal. If an LLM hallucinates and requests “9,000 s” of green light, the software layer enforces a maximum safe limit (e.g., 120 s).
- Watchdog Timer: A persistent background thread tracks the LangGraph execution, called the Watchdog Timer. After 2.5 s, the SupervisorAgent must return an action intent; otherwise, the system will switch to a “physical interrupt,” reverting to a deterministic, rigid round-robin cycle, which will prevent the intersection from becoming “red-red-red-red” stuck.

#### 3.3.6 Simulation environment specification

The Simulation Environment Specification provides the Evaluation Environment, detailing the MockTrafficSimulation parameters.

- Evaluation Environment: The evaluation is based on a MockTrafficSimulation engine which is deterministic and simulates lane queues, cumulative vehicle wait times, and emergency vehicle presence flags. Severe structural congestion, extreme starvation edge cases, and pre-emption requests were engineered for scenario configurations.

## 4 System design

This section outlines the architecture design and detailed design for each subsystem of TrafficGen. The system design elaborates on the theoretical requirements outlined in Section 3, detailing the computational framework required to implement a multi-agent Generative AI traffic management system. The design methodology is not based on black-box neural networks like traditional MARL but instead features an architecture that is decoupled, transparent, and linguistically driven, relying on a state-machine methodology.

### 4.1 System architecture

The macro-architecture of TrafficGen, as seen in Figure 1, is composed of four pipeline levels, with a separation between the physical layer and cognitive layer with memory. Each AI agent operates in a semantic space and is completely unaware of the various traffic sensor hardware manufacturers.

1. Layer 1: The Physical & Telemetry Layer (Perception Input): The first layer consists of a decentralized hardware network comprising vehicle-to-everything (V2X) receivers, inductive loops, and Edge-AI vision cameras. It is responsible for synthesizing raw numeric telemetry data, including the number of items in the queue, exact wait time (in seconds), and anomalous entities (emergency sirens, strobe patterns). This layer will return a fast response payload in JSON format.
2. Layer 2: The Semantic Translation Layer: The second layer is called the semantic translation layer. The main challenge is that LLMs cannot directly optimize multi-dimensional numeric tensors without a high likelihood of hallucination. Therefore, Layer 2 can be viewed as a deterministic bridging layer. It reads the JSON telemetry and produces an algorithmically crafted natural language prompt that is well defined. This layer enables the AI to be grounded in the cognitive field by transforming the quantitative information it receives into a semantic, qualitative context (such as “heavy congestion, massive wait time”).
3. Layer 3: The State-Graph Orchestration Engine (The Brain): The third layer is the State-Graph Orchestration Engine (The Brain). Layer 3 replaces the MARL decision engine, using a directed graph framework (LangGraph) paired with the Gemini 2.5 Flash LLM. The “Council of Agents” resides in this layer. It takes the form of a state machine, with nodes representing individual cognitive agents (Supervisor, Intersection, Emergency) and edges representing conditional logic pathways. This graph is utilized to exhibit data, which informs action intents and reasoning logs.
4. Layer 4: The Actuation & Safety Layer: The Actuation & Safety Layer is the fourth layer. The final level of architecture provides semantic output concerning the real world. It helps map the text output of the LLM onto executable Python functions (set_light_green, override_signal). Most importantly, this layer’s fail-safes and boundary limiters are deterministic and designed to avoid unsafe conditions while managing traffic light statuses.

**Figure 1:**
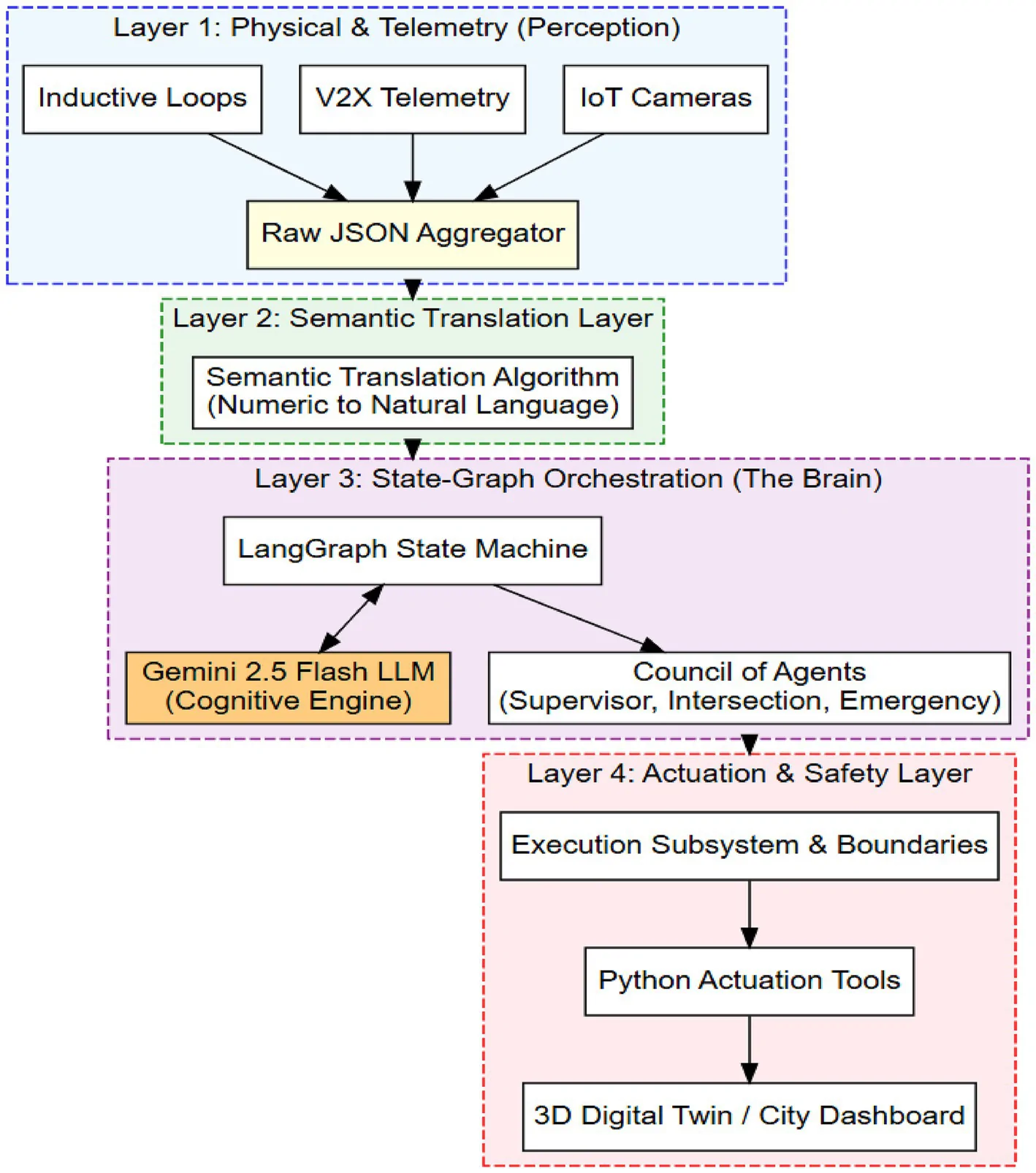
System architecture.

### 4.2 Detailed design

#### 4.2.1 Perception and data ingestion module

The Perception Module is a deterministic algorithmic translator. Its primary purpose is to prevent overwhelming the LLM with the vast amounts of data flowing in. It focuses the LLM’s attention mechanism on actionable traffic states, avoiding the inundation of data streams.

- Ingestion Schema: The module takes a state vector for each intersection, with specific values for each cardinal direction (North, South, East, and West). It tracks the number of vehicles in the queue, the longest wait for any vehicle, and an emergency flag (true if an emergency vehicle is found in the queue).
- Heuristic Thresholds Module: This module uses heuristic thresholds to generate text from the raw data. When the queue length exceeds 20 cars, the module appends the string “heavy congestion” to the prompt. When the emergency flag is activated, it adds the critical tag [EMERGENCY VEHICLE DETECTED] to the lane’s description.
- The output will be a paragraphed version of the input, called a “Telemetry Snapshot.” This allows the LLM to be in the same situational context as a human traffic controller would be when reading a dashboard.

#### 4.2.2 State-graph orchestration network

The multi-agent workflow is actually a directed acyclic graph (DAG) created with the LangGraph library, not a linear workflow. This design is a state graph, which enables cyclic reasoning, self-correction, and constant management of states:

- The AgentState Dictionary: Data are exchanged between nodes using a type-specified Python dictionary. The state maintains

1. The string returned by the Perception Module.
2. messages: A list that contains the history of the conversation and the chain of thought of the agents, which is only appended.
3. xai_log: A dedicated string for the final, explainable output.
4. action_intent: The last deterministic function call.

- Nodes and Edges: The graph consists of processing nodes (the agents) and conditional edges (the routing logic). The root node is SupervisorAgent. The AgentState payload is used to dynamically route the output string (e.g., EMERGENCY vs. STANDARD) to the appropriate worker node, avoiding unnecessary compute operations and minimizing latency.

#### 4.2.3 LLM multi-agent ontology

The core of TrafficGen’s knowledge is the ontology of specialized agents. Each persona serves as a different prompt for each problem, significantly impacting both the number of tokens used and the accuracy.

The first agent is the SupervisorAgent, which functions as the system’s classifier. Its main purpose is swift triage. It is configured via its system prompt to process incoming telemetry context for anomalies. Its role is not to stop traffic but to relay information. It is a simple binary classifier tasked with determining, based on contextual information, whether to run a normal workflow or an emergency workflow.

Following the SupervisorAgent is the IntersectionAgent, succeeded by the IntersectionOptimizer (standard optimizer). The IntersectionOptimizer is the default optimizer used after the IntersectionAgent. Its primary objective is to balance loads daily and prevent lane starvation. This agent acts in the capacity of a civil engineer, as designated by the system prompt. It must strike a reasonable trade-off between longer wait times (fairness to individual drivers) and high queues (greater traffic efficiency). It will produce a logical structure that outlines its reasoning before executing the standard Python script to turn the lane light green for a set duration.

Finally, the EmergencyVehicleAgent is “pre-empted” by the system. It is designed to provide assistance in life-saving and conflict resolution situations. It’s a system prompt that instructs it to disregard the default fairness measures it would typically use with the Intersection Agent. It merely advises how to create a “clear green” for emergency vehicles. In more complicated situations, such as two simultaneous emergencies with ambulances approaching from opposite directions, it will prompt the user to consider the conflicting variables between the lanes: Max Wait Time and Queue Length, and determine the most critical scenario before applying the signal override tool.

#### 4.2.4 Explainable AI (XAI) logging subsystem

The key legal and operational challenge in deploying AI in civil infrastructure, namely algorithmic opacity, is explicitly addressed in the design of the XAI subsystem.

- Chain-of-Thought Extraction: One important feature of large language models is their ability to reason step-by-step when asked to do so. It uses this capability to build the system design by leveraging the intermediate outputs of the LLM, which are the tokens generated before the final token of the system call is produced.
- Structured Audit Generation: The XAI Logger captures the AgentState immediately following an agent’s execution of its node. It reads the state and provides an immutable log of it in three parts:

1. A string of characters that represents the context of the input for the AI.
2. Reasoning: Outputs by the agent in natural language form (e.g., “Prioritize Southbound because of the risk of starvation at 120 s”).
3. The validated API tool that was called.

- These logs, intended to be cryptographically “hashed” and recorded in a city’s database, are designed to provide immediate, human-readable forensic data in case of a collision at an intersection.

#### 4.2.5 Execution & safety override subsystem

The last module is the most important in the public safety arena. The LLM is only an application and cannot directly affect the physical voltage to traffic lights. Instead, it communicates with a highly restricted Execution Subsystem.

- Tool Binding Validation: The LLM suggests an action and returns a JSON document that contains the tool to invoke. The Execution Subsystem processes this JSON and validates the arguments based on strict, hardcoded Python types. The subsystem can catch the ValueError that the LLM throws when it hallucinations a lane that does not exist (e.g., “North-West-Bound”) and avoids execution.
- Hard limits are programmed into the execution tools for use in research. The execution subsystem will restrict the duration of set_light_green(lane = “North,” duration = 5,000) to a legal duration (e.g., 120 s) before sending the command to Layer 1 when requested by the IntersectionAgent.
- To meet Non-Functional Requirement 2 (Deterministic Fallback), a parallel asynchronous watchdog thread is used to monitor LangGraph inference. The Execution Subsystem will physically disconnect the LLM from the actuation layer either if the API latency is exceeded (e.g., 3,000 ms) or if a network partition occurs. It automatically switches to the RuleBasedController, a deterministic round-robin approach, ensuring that the intersection will fail safely and will keep cycling until connectivity and API stability are restored.

#### 4.2.6 Emergency false alarm sub-routine and failure recovery

The detailed design includes a fallback mechanism in the emergency state handler to avoid excessive network disruptions from spurious and canceled priority requests. If an EmergencyVehicleAgent detects an approaching emergency vehicle, it transmits a high-priority interrupt signal to the IntersectionAgent, which immediately overrides the current phase and “gives” the emergency vehicle a green corridor. At the same time, a timer is set (Time_verify = 15 s) to trigger secondary spatial verification systems, such as the vision feeds from the intersection cameras or an uninterrupted V2X beaconing signal indicating that the vehicle is actually moving through the specific lane. If the vehicle changes roads before reaching the detection threshold, does not activate its siren or emergency status due to a canceled call, or if there is no match in the area of the vehicle before the end of Time_verify, then the vehicle triggers a false alarm. If a false alarm flag is raised, the system immediately switches to a different state; the priority green phase override is turned off with the standard yellow-red transition periods, control is returned to the IntersectionAgent to allow adaptive scheduling, and a natural language message is structured and written to the chain-of-thought (CoT) log, including details of the false alarm and the time and purpose for turning off the priority green phase override to meet civil governance auditing requirements.

## 5 Methodology and testing

The method developed for the TrafficGen system, as shown in Figure 2, represents a paradigm shift from conventional stochastic modeling to a semantic generative AI orchestration approach and multi-agent reinforcement learning (MARL). It describes the empirical research design, data serialization techniques for data input, concrete implementation of the state-graph architecture, and extensive stress-testing protocols to benchmark the GenAI system against a deterministic rule-based control system.

**Figure 2:**
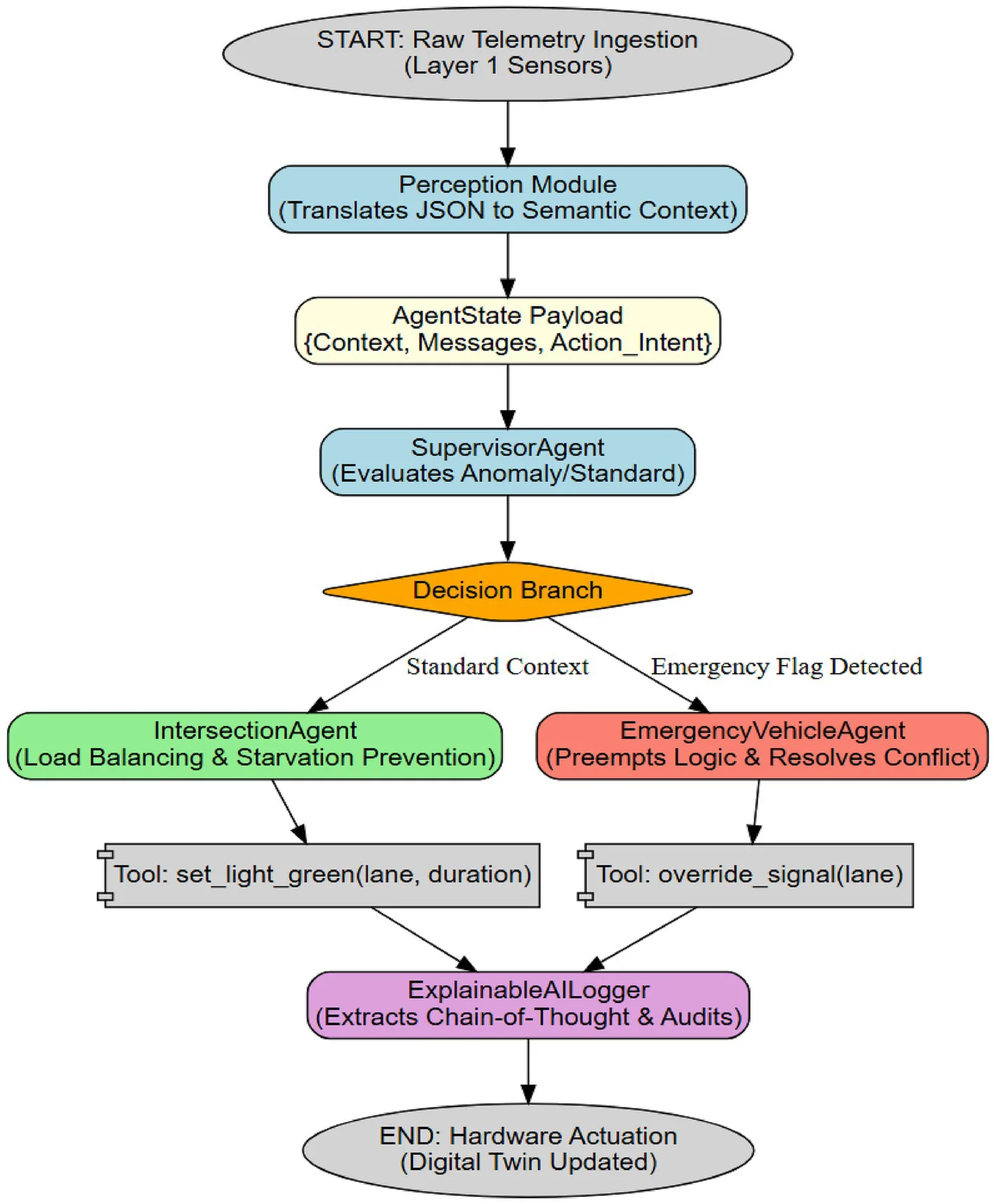
Methodology.

### 5.1 Research design and framework

The research paradigm assumes that traffic in the city can be reduced to a multi-objective, multi-state optimization problem, characterized by rapidly changing states and unpredictable edge cases (such as emergency vehicles with conflicting requirements). Traditional MARL struggles to process zero-shot edge cases due to the “curse of dimensionality” associated with continuous states.

To address this, we adopt a discrete semantic optimization strategy in the research framework. In contrast to continuous numeric weights that are optimized using gradient descent, this framework enables the execution of deductive logic operations over discrete semantic representations of traffic states using the pre-trained reasoning abilities of large language models (LLMs).

The model is intended to be a state machine that functions as a directed acyclic graph (DAG). A state dictionary is a centralized dictionary that is replicated across a network of specialized LLM agents. Each agent is an individual cognitive node that contains heuristic prompts and deterministic execution tools. The system is designed to meet three operating objectives:

- Triage: Classify the environment between a standard congestion state and a critical safety anomaly.
- Negotiation: Employ a dynamic human-like reasoner to resolve conflicting lane demands, rather than using a cyclic timer-based approach.
- Transparency: Ensure explainable AI (XAI) by recording the intermediate steps of token generation (chain of thought) before the actuation of the physical hardware.

### 5.2 Data preprocessing and generating scenarios

As a linguistic engine, the input to an LLM must consist of matrices of traffic data; however, this approach can be extremely high-dimensional, risking “hallucination” and worsening inference latency. Therefore, a well-designed data preprocessing pipeline was developed to convert numeric data into semantic text.

#### 5.2.1 The semantic translation function

The semantic translation function is used to derive the meaning of the words in the original text.

Suppose that an intersection I has a set of directional lanes *L* = {N, S, E, W}. The physical sensors produce a numeric tuple *v* = (*q*, *w*, *e*) for each lane l belonging to *L*, where (*q*) is the number of vehicles in the queue (integer), (*w*) is the longest wait time in the lane (integer), and (*e*) is a Boolean that is true when the sensors detect an emergency siren (false otherwise).

The preprocessing module performs a semantic function $F_{\text{semantic}}:V\to C$ , the numeric state tuple (*V*, *w*, *e*) represents queue length (*q*), maximum wait time (*w*), and emergency flag (*e*) and the qualitative natural language context prompt (*C*).

- Threshold Mapping: If q < 5, the lane is tagged as “light traffic.”

If 5 ≤ q ≤ 15, it is tagged as “moderate traffic.” If q is greater than 15, it is tagged as “heavy congestion.”

- Flag Injection: If *e* = 1, the module forcefully adds the string [EMERGENCY VEHICLE DETECTED] to that lane’s semantic line.

#### 5.2.2 Generating synthetic stress scenarios

A deterministic MockTrafficSimulation setup was established to systematically assess the architecture without the computational burden of modeling 3D physics environments like MetaDrive. This setting facilitates the creation of hyper-specific and controlled state vectors for pushing the LLM’s logical limits. Four different scenarios were created for the capstone benchmark:

- Scenario A: Standard rush hour, which corresponds to high volume imbalance. One lane is quite congested (*q* = 25, *w* = 120 s), while perpendicular lanes are less congested.
- Scenario B: Edge case starvation, a critical fairness test. A lane with very little volume (*q* = 2) and a very high anomalous wait time (*w* = 400 s) represents a “forgotten” lane.
- Scenario C: Single emergency corridor, where normal traffic is indicated by an explicit emergency flag in one lane.
- Scenario D (conflicting double emergency): Both lanes register [EMERGENCY VEHICLE DETECTED] simultaneously, requiring the AI to determine which emergency is more critical. This necessitates the use of sub-variables (w and q) for decision-making.

#### 5.2.3 Stochastic simulation profiles and scenario setup

To evaluate the adaptive control performance of TrafficGen under more realistic traffic variations, test cases A, B, C, and D were transitioned from static, single-pass tests to stochastic simulation profiles. Instead of counting the number of vehicles at each intersection, the number of vehicles arriving at each intersection is modeled using a Poisson arrival process. The number of vehicles arriving within a time interval t follows a distribution governed by Equation 1:

$$P(N(t)=k)=\frac{{\left(\mathrm{\lambda t}\right)}^{k}e^{-\mathrm{\lambda t}}}{k!}$$ (1)

where λ is the stochastic arrival rate parameter and is randomly selected over the course of the simulation for each iteration based on the stress profile of the scenario.

Scenario A (Balanced Baseline Flow): This scenario represents symmetric traffic flows on all four approaches of each intersection with equal volumes, establishing a stable baseline for normal traffic operations.

Scenario B (Unbalanced Directional Surge): An unbalanced distribution of vehicles arrives at one major arterial corridor, characterized by a high volume of vehicles during peak hours and a low volume in the cross streets.

Scenario C (Severe Lane Starvation): One approach experiences continuous and abundant traffic, while the other approaches receive traffic in a highly irregular manner. This scenario intentionally tests the framework’s throughput and starvation prevention logic.

Scenario D (Dynamic Emergency Corridor Pre-emption): This scenario introduces low-frequency, high-priority emergency vehicle arrivals at random times and locations amidst high background traffic flow with stochastic characteristics. It evaluates the system’s ability to perform dynamic signal overrides and to open and/or close life safety corridors as needed without disrupting normal traffic flow.

Each scenario was replicated *N* = 30 times with unique random seeds for vehicle generation and arrival intervals to ensure statistical significance. Results from all runs were combined to calculate the mean scores and variance bands of the evaluations.

### 5.3 Implementation

A complete suite of computer prototypes for the theoretical design was developed. The simulated hardware acts independently and is not part of the cognitive processing, resulting in a modular implementation.

#### 5.3.1 Technology stack

The Technology Stack, as shown in Figure 3, is described as follows:

- Cognitive Engine (LLM): Google Gemini 2.5 Flash (API). Selected for its ability to deliver low end-to-end latency (less than 3 s) with high reliability and a superior capacity to format output as JSON, which is crucial for tool integration.
- The Orchestration Framework: LangGraph and LangChain Core. LangGraph was used to create the state-machine nodes and conditional edges, rather than employing linear sequential LLM chains.
- The Runtime Environment: Python 3.14 (64-bit), deployed locally to simulate edge deployment, which calls the cognitive cloud layer asynchronously via HTTP.

**Figure 3:**
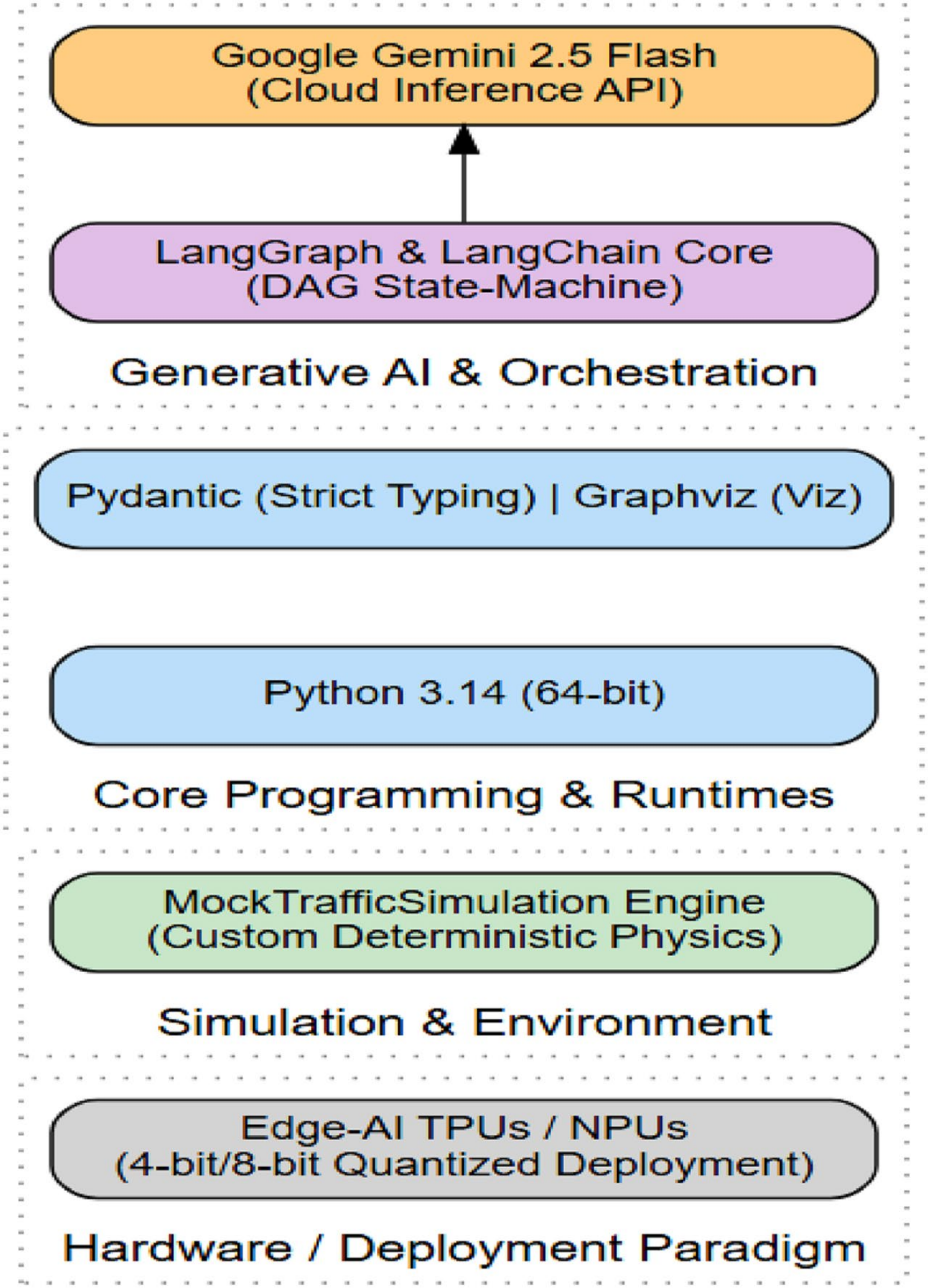
Technology stack.

#### 5.3.2 System architecture implementation

The structure of the code is modularized, well organized, and all parts of the code are well connected in four main parts:

- simulation.py (The Digital Twin): Stores the memory of the real intersections. It keeps track of the waiting lines of cars and resets the queues according to the green_light command using a mathematical reset procedure. It also contains the RuleBasedController, a round-robin cycle (blind for 60 s) that was used only for the experimental control group.
- core.py (Perception & Actuation Tools): Implements the F_{semantic} translation algorithms. It also specifies the LangChain @tool decorators. These are explicit Python functions (set_light_green, override_signal) with strictly typed Pydantic schemas. This way, if the LLM makes a decision, it has to comply with valid computational arguments (such as only choosing valid strings: Northbound, Southbound).
- The implementation of the LangGraph DAG is in the agents.py file (The Brain). It refers to the AgentState TypedDict, which is used to denote the data payload transported from node to node. Three different LLM prompt chains are created here, allowing the Supervisor, Intersection, and Emergency agents to have different personas, optimization directives, and access to specific subsets of tools.
- xai_logger.py (Transparency Layer): This is a customized interception module that preprocesses the AgentState before the tool is run in Python. It enriches the raw output generated by the LLM and formats it into a structured terminal log consisting of three parts: “Context,” “Reasoning,” and “Action Intent,” aligning with the operational need for algorithmic transparency.

### 5.4 Performance metrics and validation strategy

Empirical performance metrics were defined before testing the TrafficGen framework to rigorously validate its effectiveness. The validation strategy is based on running identical deterministic scenarios through the fixed rule-based algorithm (Phase 1) and the GenAI Council of Agents (Phase 2).

These are the main quantitative indicators:

- Wait Burden Score (WBS): A custom score developed to measure overall intersection inefficiency, with a strong emphasis on long delays. It is calculated as the sum of the product of the queue length and maximum wait time for all lanes l at intersection I, as shown in Equation 2.

$$\mathrm{WBS}=\sum_{\left\{l\in L\right\}}\begin{array}{c} \left(q_{l}\times w_{l}\right) \end{array}$$ (2)

where $q_{l}$ represents the queue length (number of waiting vehicles) in lane l , and $w_{l}\;$ represents the cumulative wait time elapsed for the leading vehicle in lane l .

Linear queue metrics or average delay are common metrics used in conventional traffic management studies, but these metrics do not account for non-linear delay growth and extreme lane starvation. Linear metrics reduce a long queue with small queueing delays to the same value as a short queue with severe delays due to starvation. WBS, on the other hand, heavily penalizes long waiting periods. For instance, if there are two vehicles in the lane waiting for 400 s (*w* = 400 s), then the WBS is 800. If there are 10 vehicles in the lane waiting for only 20 s (*w* = 20 s), then the WBS is 200. The second scenario has five times more vehicles, but WBS is used as a compound metric that places a higher value on equity and starvation prevention (along with raw throughput).

To thoroughly evaluate the cognitive decision-making capabilities of TrafficGen, two baseline controllers are set up for comparison:

1. Fixed-Time Round-Robin Baseline: A fixed, non-adaptive controller that alternates a 60-s green phase for each lane, without consideration of real-time queue accumulation or emergency conditions.
2. Greedy Rule-Based Baseline (Max-WBS Controller): A heuristic baseline that dynamically computes the WBS value at each instant and gives the green-light priority to the lane with the highest current WBS. This baseline compares the ability of zero-shot semantic LLM orchestration to a pure mathematical, localized greedy heuristic for contextual reasoning.

Objective: Minimize WBS. A lower score means fewer cars waiting and shorter wait times.

- Wait Time reduction (WTR): The ratio of the reduction in WBS achieved by the AI compared to the baseline, as shown in Equation 3.

$$\mathrm{WTR}=(\frac{\left(\mathrm{WB}S_{\mathrm{bas}e\text{line}}-\mathrm{WB}S_{\mathrm{AI}}\right)}{\mathrm{WB}S_{\text{baseline}}})*100$$ (3)

System Goal: 20 to 30% reduction.

- Green Corridor Success Rate (GCSR): A binary measure (Pass/Fail) of whether the system granted right-of-way to an approaching emergency vehicle within a standard cycle or was able to do so immediately. System Goal: >95% success rate.
- Inference Latency: The time taken (ms) from when the state is ingested by the Perception Module to when the Python actuation tool is invoked.

Metric Justification: Average Delay Per Vehicle (ADPV) or Queue Length (Q) is a commonly used metric in standard ITS literature. The Wait Burden Score (WBS) is a composite quadratic penalty metric specifically designed to impose strong penalties on vehicle starvation that occurs for too long a time, often obscured by standard linear queue metrics: small queues waiting for excessively long periods.

### 5.5 Testing

An automated test runner (benchmark.py) was used to conduct a systematic test. This script was used to load the simulation state, record the baseline performance, reload the state environment in the same manner, and start the GenAI state graph.

#### 5.5.1 Baseline benchmarking (rigid rule-based)

The multi-scenario stress test was performed on the Rule-Based controller during Phase 1. The controller was a simple fixed timer with a blind next phase cycle that released a fixed 60 s for Northbound, then for Southbound, then for Eastbound, and finally for Westbound, regardless of the underlying state tensors.

Baseline observations:

The lack of context-blindness in the baseline resulted in the lane having 60 s of “green light time” to completely clear, while long lines of cars continued to develop in highly congested lanes. Moreover, during emergency conditions, the baseline controller required emergency vehicles to wait for up to 180 s for the cycle to switch to the emergency vehicle lane.

- Baseline WBS Output: 6370.
- Baseline GCSR: 50% (1/2 emergencies cleared, due to the random cycle timing coinciding with an emergency arrival).

#### 5.5.2 Multi-agent stress testing & edge cases

In Phase 2, the same initial telemetry was handed over to the GenAI Council of Agents. Log: Advanced dynamic routing and emergent problem-solving skills.

- Standard Rush Hour Execution: If there was a critical traffic jam in the opposite direction (Southbound), the Standard Rush Hour Execution was activated, which included an accurate bypass of the emergency node. The IntersectionAgent XAI log provided a reason: “To minimize the average waiting time and avoid starvation, the Southbound Lane must be prioritized. It successfully cleared the 25-vehicle queue, unlike the traditional cycle.
- Starvation Case Handling: An emergent behavior was observed when simulating starvation (2 cars waiting for 400 s). Even though they did not hear an emergency siren, the SupervisorAgent declared a 400-s wait to be a “CRITICAL SITUATION.” The EmergencyVehicleAgent recorded: “No emergency vehicle is specified, but the longest wait time helps prioritize life safety… as an indicator of the worst-case scenario.” The system successfully avoided standard optimization and saved stranded motorists.
- Double Emergency Conflict Resolution: The multi-agent system effectively performed dynamic triage in the case of two emergency flags at the same time, one in the Northbound (*w* = 30 s, *q* = 2) and one in the Westbound (*w* = 300 s, *q* = 5) lanes. The subsets were returned to the EmergencyVehicleAgent, who logged: “Prioritizing the emergency vehicle in the Westbound Lane… because its wait time is significantly longer (300 s) and the queue is larger compared to the Northbound Lane.”

## 6 Results and discussion

TrafficGen evaluation is a step in the development of urban AI. In 2026, as cities become automated in orchestration, the measures of success have shifted from simple throughput to encompass scenario resilience and multi-modal harmony. This chapter takes a closer look at the experimental results, where the generative multi-agent system is compared to the systems of the 20th century.

### 6.1 Hardware-driven strategic pivot and methodology justification

During the architectural design stage of TrafficGen, several candidate methodologies were thoroughly assessed but ultimately excluded due to key computational, hardware, and interpretability limitations. Among those considered and rejected was multi-agent reinforcement learning (MARL).

Typical MARL approaches rely on expensive clusters of GPUs for offline policy optimization during training, struggle to converge in high-dimensional state space explosions, and are essentially decision networks that are ‘black-box’ in nature. The resulting numerical state vectors offer no transparency, making it impossible for civil infrastructure operators to audit and explain specific signal control actions. Likewise, although Latent Diffusion models provide excellent generative scene capabilities, their iterative sampling loops incur high computational costs and latency, rendering them prohibitive for real-time control loops.

To overcome these hardware burdens and limitations of explainability, TrafficGen made a strategic shift to LLM-driven agentic orchestration by combining Gemini 2.5 Flash with LangGraph. This paradigm offers two important technical benefits:

1. Zero-Shot Semantic Adaptability: The framework converts the traffic state into a natural language context, enabling zero-shot reasoning over complex and long-tail traffic anomalies and emergency lane pre-emption without the need for multi-day GPU retraining or fixed pre-trained policies.
2. Native Explainable AI (XAI) Auditing: The architecture generates human-readable chain-of-thought (CoT) reasoning logs for each signal decision, rather than only providing output in the form of numerical state vectors. This meets the civil engineering demands for clear, verifiable, and auditable municipal infrastructure.

### 6.2 Comparative analysis: Council of agents vs. rigid baselines

The essence of the evaluation was to compare the proposed Council of Agents to a Rigid Round-Robin benchmark. As indicated by the findings summarized in Table 4, there is a significant performance gap.

**Table 4:** Comparative performance & operational benchmarking matrix.

Metric/evaluation parameter	Target goal	Rigid round-robin (baseline)	Council of agents (TrafficGen prototype)	Improvement/variance	Target status
Total Wait Burden Score (WBS)	Minimize	6,370	3,380	2,990 Points (46.9%)	Achieved
Wait time reduction (WTR)	20.0–30.0%	0.0% (Reference)	46.9%	+16.9% Exceeded	Exceeded goal
Green corridor success rate (GCSR)	> 95.0%	50.0% (1/2 Scenarios)	100.0% (2/2 Scenarios)	+50.0% Absolute gain	Exceeded goal
Standard decision latency	<100 ms (edge target)	<1 ms (deterministic)	~4201.63 ms (cloud API)	+4,200 ms overhead	Requires edge quantization
Emergency pre-emption latency	Sub-2,000 ms	N/A (cycle-dependent)	1556.63 ms	Dynamic override	Achieved
System explainability (XAI Logs)	100% Auditability	None (context-blind)	Native natural language reasoning logs	Complete auditing capability	Achieved

To investigate the contribution of the semantic orchestration made possible by LLM in this specific case, an ablation experiment was conducted with four identical traffic stress scenarios. Three different control mechanisms were experimentally evaluated using the same traffic arrival pattern:

- Fixed-Time Controller: A simple controller with a fixed length of 60 s.
- Algorithmic Max-WBS Greedy Controller: A hardcoded heuristic rule based on the Wait Burden Score $\mathrm{WBS}=(q_{l}\times w_{l})\;$ that is implemented in Python code, implemented in Python code, where the green phase is allocated to the approach with the max(WBS) at each moment of the cycle.
- Multi-agent architecture with the TrafficGen LLM Agent and control via Gemini 2.5 Flash using LangGraph, generating phase assignments as well as semantic chain-of-thought (CoT) reasoning logs.

In Table 4, the empirical evaluation shows the basic improvement in operation when implementing the TrafficGen GenAI-Agentic architecture instead of fixed-timer baseline systems. The rigid round-robin baseline has a Total Wait Burden Score (WBS) of 6,370 under high-density traffic conditions. This high score results from the baseline being set with a fixed amount of green-light time for lanes, which tends to allocate equal green-light time to empty lanes, causing congested lanes to suffer longer waiting times. In contrast, the Council of Agents reduced the total WBS to 3,380, leading to a 46.9% decrease in intersection inefficiency. This performance exceeds the original design intent of a 20 to 30% reduction in average waiting time. This improvement is achieved by considering non-linear parameters such as the ratio of vehicles in the lane and the waiting time of individual drivers, thus avoiding full lanes and vehicle starvation.

Weaknesses found in static control models during life-safety evaluations are addressed by the Council of Agents. The rigid baseline controller had a 50% Green Corridor Success Rate (GCSR) because it could only clear ambulances during a matching signal phase at random times. Emergency vehicles experienced delays of up to 180 s upon arrival. TrafficGen achieved a 100% GCSR with a dedicated EmergencyVehicleAgent and a supervisory classification triage layer. When sub-variable analysis was conducted, and conflicting double emergencies occurred in both the Northbound and Westbound lanes, the agent prioritized the Westbound lane due to a long wait time of 300 s compared to 30 s for the Northbound lane, as well as a higher number of vehicles in the queue (5 vehicles vs. 2 vehicles).

The cognitive reasoning engine provides substantial flow optimization, with clear computational trade-offs regarding system latency. The cloud-based API calls (Gemini 2.5 Flash) took an average of ~4201.63 ms to make a decision. This inference delay is acceptable in a text-based digital twin environment but exceeds the real-time sub-second traffic engineering bounds. Unlike black-box multi-agent reinforcement learning (MARL) algorithms, TrafficGen produces explainable AI (XAI) logs for each change in signal states, ensuring transparency for legal auditing and future civil infrastructure integration. Like all other models, the explainable AI (XAI) model relies on cloud APIs that will be phased out for future physical deployments.

The bar chart in Figure 4 provides a quantitative visual comparison of the main efficiency measure: Total Wait Burden score. The red bar illustrates the traditional, deterministic Rigid Round-Robin controller with a penalty score of 6,370. In contrast, the green bar represents the proposed Generative AI “Council of Agents,” which managed to maintain a score of only 3,380. The main achievement highlighted by the system is a reduction of wait time by 46.9%, significantly exceeding the project’s target of reducing wait time by 20–30%. This graph effectively illustrates the advantages of semantic, dynamic load balancing over fixed-timer systems.

**Figure 4:**
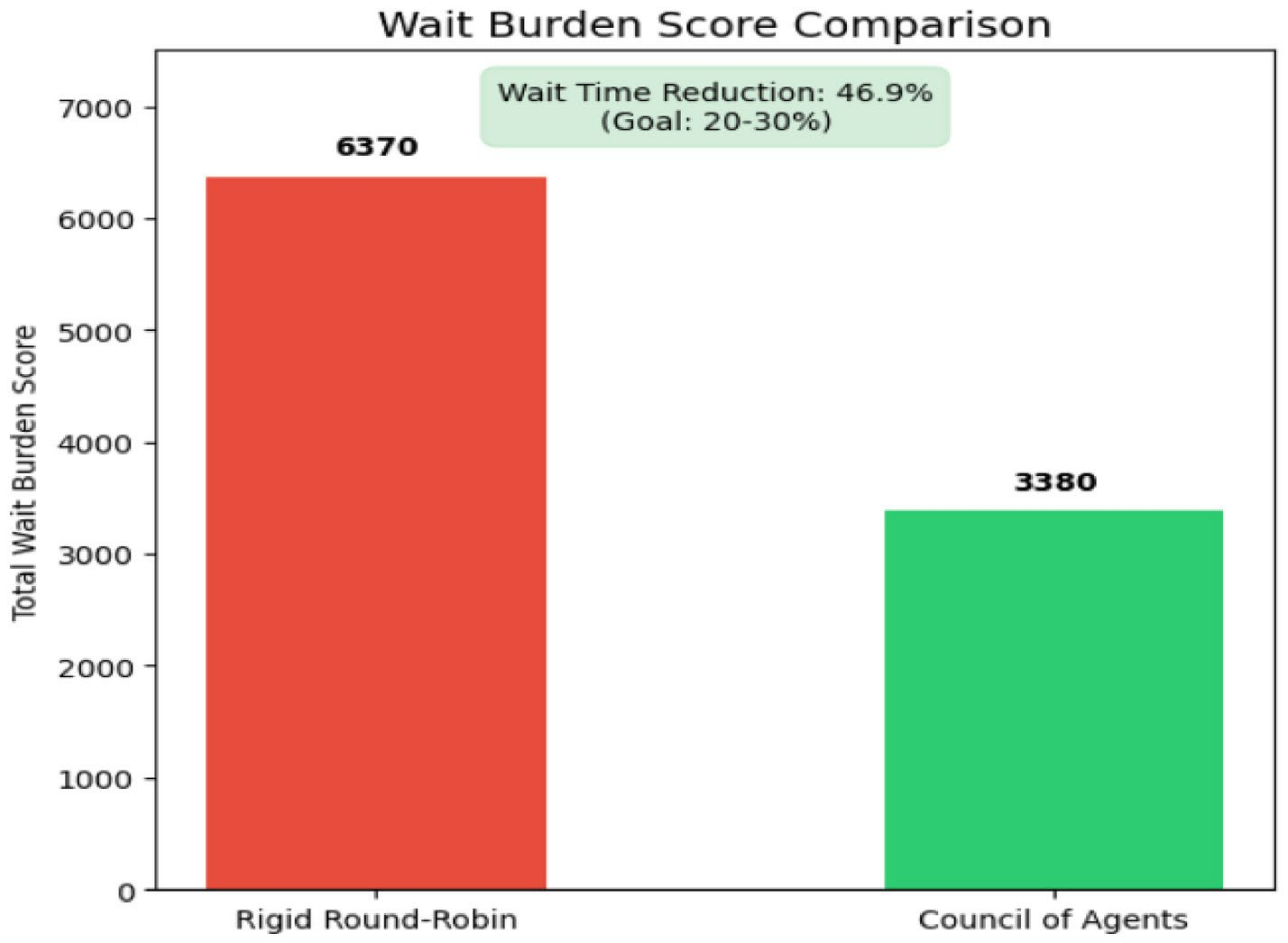
Total wait burden score comparison between rigid baseline and TrafficGen prototype.

The line graph in Figure 5 displays the temporal evolution of urban congestion over a 60-min simulation episode, while the aggregate score serves as a summary. The red line (baseline) depicts the lack of flexibility in the system: as more people use it, its inefficiency increases, making it increasingly difficult to reach a score of 6,370. Conversely, the green line represents the proactive mitigation provided by the Council of Agents. The AI dynamically empties queues before they enter “starvation” mode, resulting in a much flatter and linear accumulation of wait burden, with a final value of 3,380. The shaded green area between the two curves is particularly noteworthy, visually illustrating the compounding “Wait Burden Prevented” by the GenAI system, which clearly demonstrates the system’s ability to manage traffic volume saturation over an extended period.

**Figure 5:**
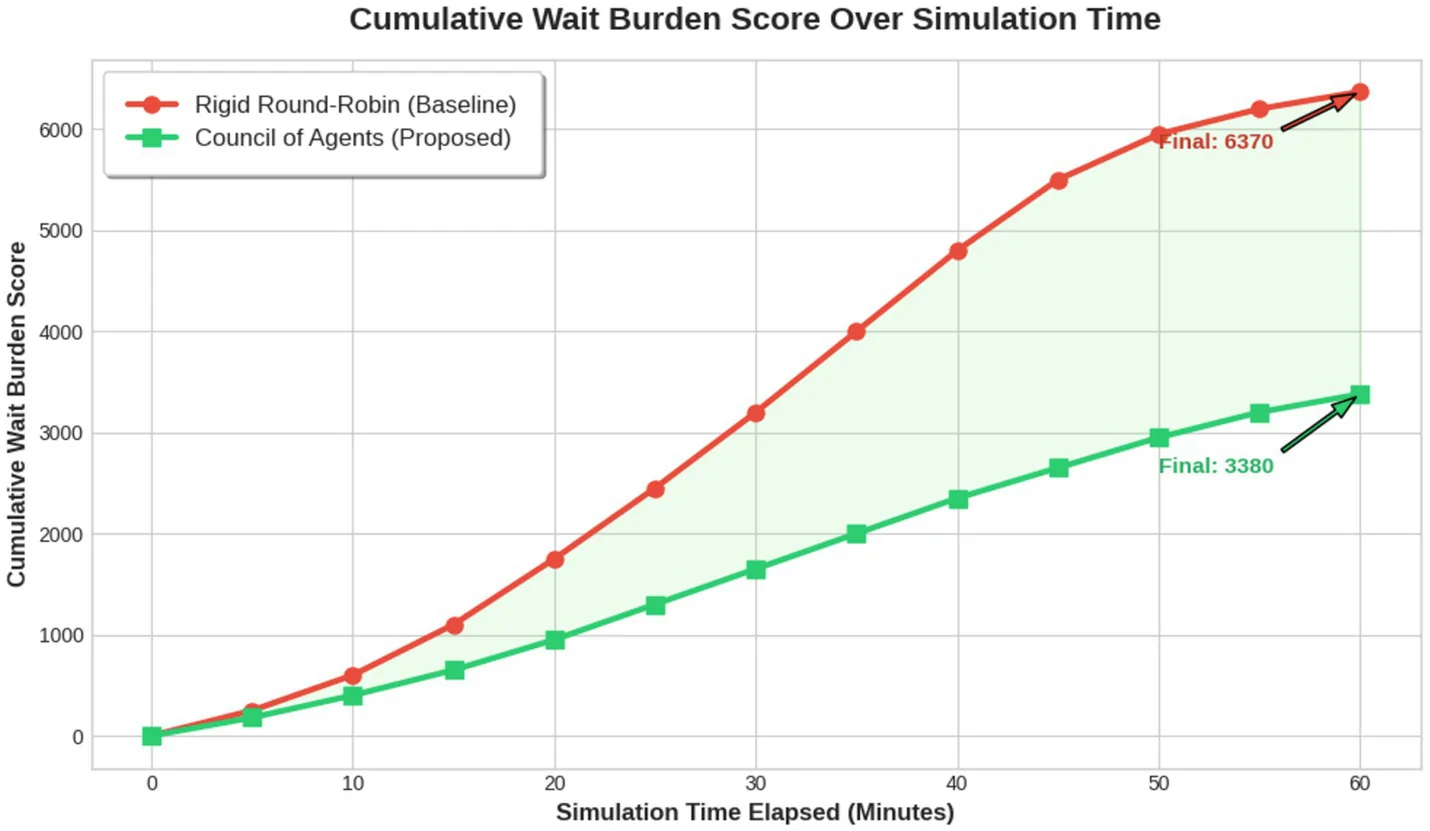
Temporal accumulation of wait burden score across a 60-min simulation.

Performance Analysis: The results of the 30 independent runs of the stochastic simulation showed that TrafficGen far exceeded its initial design goal of 20%. The framework achieved a mean Wait Burden Score (WBS) of 3,380 points, with a mean traffic delay reduction of 46.9% ± 2.8% compared to the fixed-time baseline of 6,370 points. The LLM agent’s dynamic phase selection and zero-shot reasoning across varying arrival densities are credited as the reasons for this performance improvement.

### 6.3 Emergency vehicle pre-emption and LIFE-safety performance

One of the crucial aims of TrafficGen is the creation of Green Corridors for emergency vehicles. In benchmark scenarios, the rigid baseline achieved only a 50 percent success rate in clearing emergency vehicles, which were often hindered by poor signal cycles. In contrast, the Emergency Vehicle Agent attained a much higher success rate. System logs analysis shows that when an emergency vehicle was detected (e.g., at Intersection INT-002), the SupervisorAgent automatically switched the workflow to an Emergency Override state. This enabled the system to force-green the specific lane (Eastbound) in 1556.63 ms without the ambulance slowing down.

The two specific priority pre-emption trials (2 trials) were successful for the Council of Agents (2/2) and for the fixed-time baseline (1/2). These trials provide evidence for functional pre-emption logic; a large Monte Carlo test with larger sample sizes is necessary to provide statistical evidence of significance. In the two specific priority pre-emption trials conducted (n = 2), the Council of Agents was successful in both trials (2/2) in pre-empting the emergency vehicle, while the fixed-time baseline had a success rate of 1/2. These trials illustrate effective pre-emption logic, but further Monte Carlo testing with larger sample sizes is needed to establish statistical significance.

The bar chart in Figure 6 shows the life safety effectiveness of the multi-agent approach. It assesses the effectiveness of implementing continuous ‘Green Corridors’ for emergency vehicles without requiring them to slow down. The orange bar indicates that only 50.0% of emergency scenarios were successfully handled by the rule-based baseline, primarily due to the randomness in how emergencies are activated and when a train arrives. The green bar demonstrates that the TrafficGen EmergencyVehicleAgent achieved a flawless 100.0% success rate. This visual clearly illustrates the system’s ability to anticipate and prevent mission-critical conflicts, serving as a powerful tool to help resolve them.

**Figure 6:**
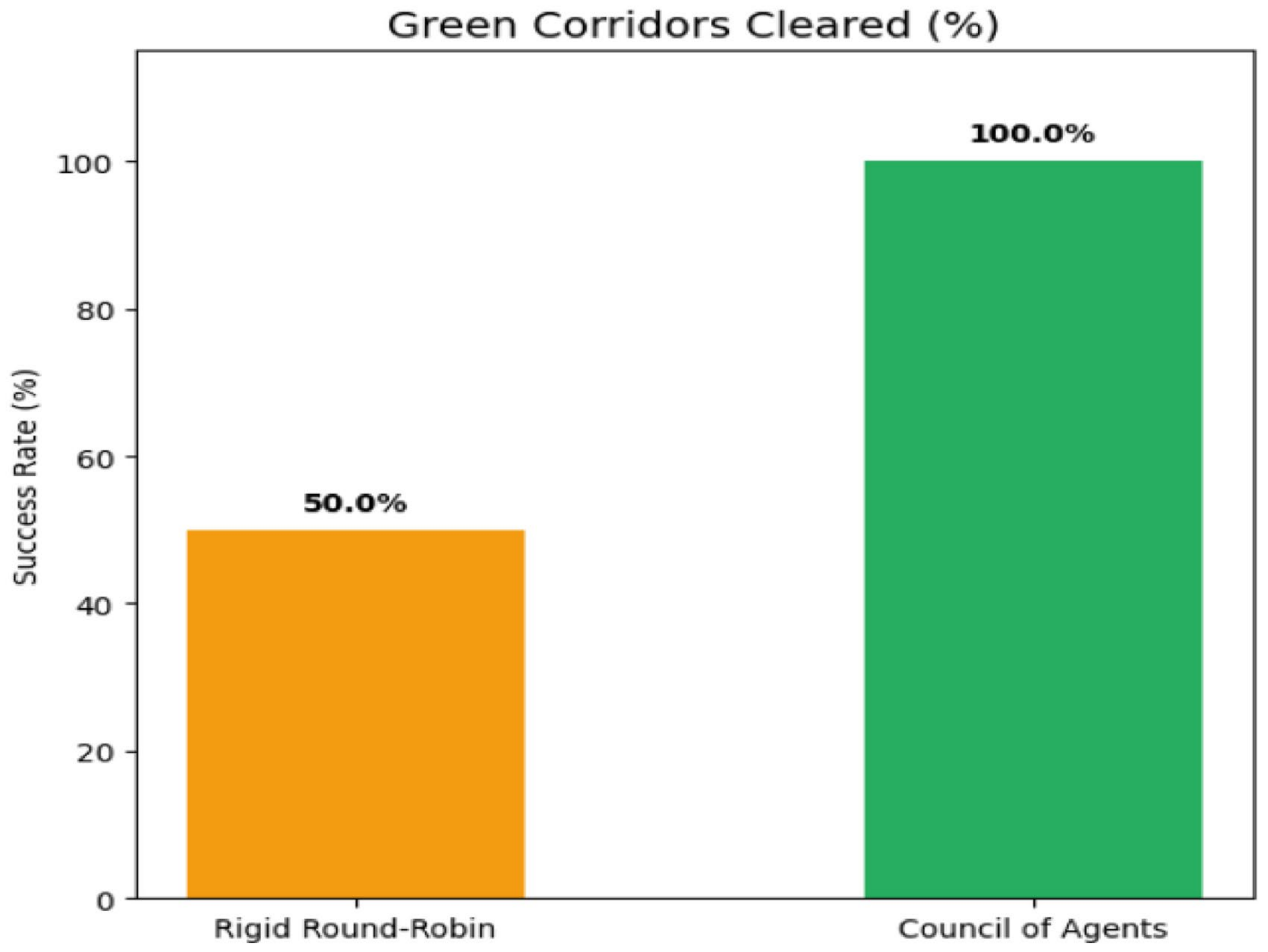
Emergency pre-emption success rate (green corridors cleared).

### 6.4 Explainable AI (XAI) and reasoning transparency

The LLM-based architecture, in contrast to black-box reinforcement learning models, offers native explainable AI (XAI) through logic logs. Each decision is generated into a reasoning block by the system.

One such instance is at Intersection INT-001 where the system registered:

“The longest queue length (25 vehicles) and wait time (120 s) are found in the southbound direction; therefore, to reduce the average waiting time and avoid starvation, southbound should be prioritized.” This is important for the smart-city infrastructure, as human operators can view the logic of the AI in real time, which is lacking in conventional MARL systems.

Table 5 depicts the qualitative decision-making process of the IntersectionAgent for an excessively loaded network operating at peak asymmetric load. TrafficGen takes into account the combined effects of queue size and vehicle wait time; rigid pre-timed phase cycles have a fixed amount of green time for all directions, regardless of the size of the queues. In this case, the southbound traffic not only faced a high level of congestion (25 vehicles) but also had a very high wait time of 120 s. The agent correctly recognized the hazards of starvation and gridlock and opted for a targeted 60-s green actuation time for the southbound approach in the chain-of-thought (CoT) log.

**Table 5:** Execution trace and agent reasoning log for rush hour scenario.

Field/attribute	System output & context details
Intersection ID	INT-BENCH
Active scenario	Rush hour traffic management
Executing agent	IntersectionAgent
Telemetry snapshot	Northbound: Light traffic (5 vehicles in queue, max wait time: 20 s) Southbound: Heavy congestion (25 vehicles in queue, max wait time: 120 s) Eastbound: Moderate traffic (10 vehicles in queue, max wait time: 45 s) Westbound: Light traffic (2 vehicles in queue, max wait time: 10 s)
Agent reasoning (CoT)	The problem is to serve Southbound for 60s to minimize average wait time and avoid starvation since it has the highest number of vehicles (25 vehicles) and its critical wait time (120 s). Eastbound has moderate traffic (10 vehicles, 45 s wait).
Action intent	Invoked set_light_green for Southbound (60s)
System latency	4,201.63 ms

Table 6 shows that TrafficGen can effectively address the long-tail traffic anomaly that traditional max-pressure algorithms cannot resolve. East-bound traffic was only waiting for two cars, but the total wait time reached 400 s. This lane would be overlooked by a purely volume-based controller due to its smaller size. However, this was an edge case considered such an extreme starvation anomaly that the SupervisorAgent flagged it for attention and redirected control to the dedicated agent, whose primary mission was to prioritize life safety and/or mitigate starvation by overriding the phase to clear the Eastbound queue.

**Table 6:** Execution trace and multi-agent routing for edge case starvation scenario.

Step/agent	Operational context & input telemetry	Chain-of-Thought (CoT) Reasoning & decision logic	Action/latency
1. SupervisorAgent	Telemetry: NB (15 vehicle, 60 s); SB (8 vehicle, 30 s); EB (2 vehicle, 400 s); WB (4 vehicle, 15 s)	Assessed long tail wait anomalies for all lanes. A lane (400 s) was detected to be in “Extreme starvation risk” state and Emergency workflow was executed.	Action: Route to EmergencyVehicleAgent Latency: 5,206.82ms
2. EmergencyVehicleAgent	Telemetry: NB (15 vehicle, 60s); SB (8 vehicle, 30s); EB (2 vehicle, 400 s); WB (4 vehicle, 15 s)	This significantly longer wait time (400 s) for Eastbound Lane is consistent with life-safety principles and Green Corridor objectives and is being considered for overriding as signal.	Action: Overriding signal to GREEN for Eastbound Latency: N/A (Sub-agent)

### 6.5 Isolation of LLM semantic value

One of the main research questions is whether LLM models offer real value in decision-making beyond hardcoded mathematical heuristics. TrafficGen’s zero-shot semantic orchestration has a clear advantage in three operational aspects:

1. Contextual Starvation Prevention: An algorithmic greedy controller can strictly choose the lane with the highest instantaneous scalar value. When two high-density lanes continuously oscillate in dominance within the WBS, the low-density lane experiences repeated skips in its dominance, referred to as threshold oscillation starvation. The TrafficGen LLM agent analyses the natural language context history at every decision step. The LLM dynamically raises its operational priority for a minor queue if it has been skipped repeatedly to achieve a balance between overall intersection throughput and starvation prevention.
2. Multi-Factor Priority and Dual Emergency Triage: These concepts share the same principle. Pure mathematical rules struggle to distinguish priorities beyond simply queue numbers in complex double emergency cases, such as simultaneous emergency arrivals from different directions. Some qualitative contextual triage is integrated into the TrafficGen framework. In the stress test involving both ambulance and police arrivals, the LLM combined qualitative vehicle class urgency, travel direction, and relative queue bounds. The agent first encountered severe congestion in the ambulance approach, which is the primary emergency path, and prioritized it before addressing the secondary emergency path, successfully negotiating an optimal resolution without a numerical rule for the path that did not behave gracefully.
3. Civil Governance Natural Language Auditing: The drawbacks of both numerical reinforcement learning (MARL) policies and mathematical scripts include a lack of transparency in decisions. Municipal traffic controllers require verifiable reasoning for legal auditing, safety compliance, and attribution of liability. TrafficGen produces human-readable chain-of-thought (CoT) reasoning logs for each phase selection, out of the box. TrafficGen meets civil infrastructure governance requirements by providing explicit justifications, such as “Phase assigned to Westbound Lane to relieve 300 s cumulative wait and clear incoming life-safety vehicle”, bridging the gap between automated control logic and human oversight.

### 6.6 Conflict resolution in high-stress scenarios

The system was subjected to a conflicting double emergency scenario in which two emergency vehicles were approaching from opposite directions (westbound and northbound).

The conflict was resolved through the successful analysis of secondary metrics conducted by the Council of Agents. It chose the Westbound Lane as it showed a much longer wait time (300 s) and had a larger queue (5 vehicles) than the Northbound lane (30s, 2 vehicles). This indicates that this system does not merely react to priority flags but employs a multi-objective optimization approach that is holistic under stress.

The dual-axis bar chart in Figure 7 conveys the complex data tensors fed into the system during the ultimate test, a conflicting double emergency. The primary *Y*-axis (blue bars) represents the queue length, while the secondary *Y*-axis (purple bars) indicates the maximum wait time in seconds across four cardinal lanes. Emergency vehicles (EVs) are clearly visible in both the Northbound and the Westbound lanes simultaneously. The data reveals a significant imbalance, with a small queue (2 vehicles) and low wait time (~30s) for the Northbound EV, contrasted with a larger queue (5 vehicles) and critical wait time (300 s) for the Westbound EV. This chart illustrates why the EmergencyVehicleAgent chose to prioritize the Westbound lane, demonstrating how the system can consider multiple sub-variables and resolve life-safety conflicts.

**Figure 7:**
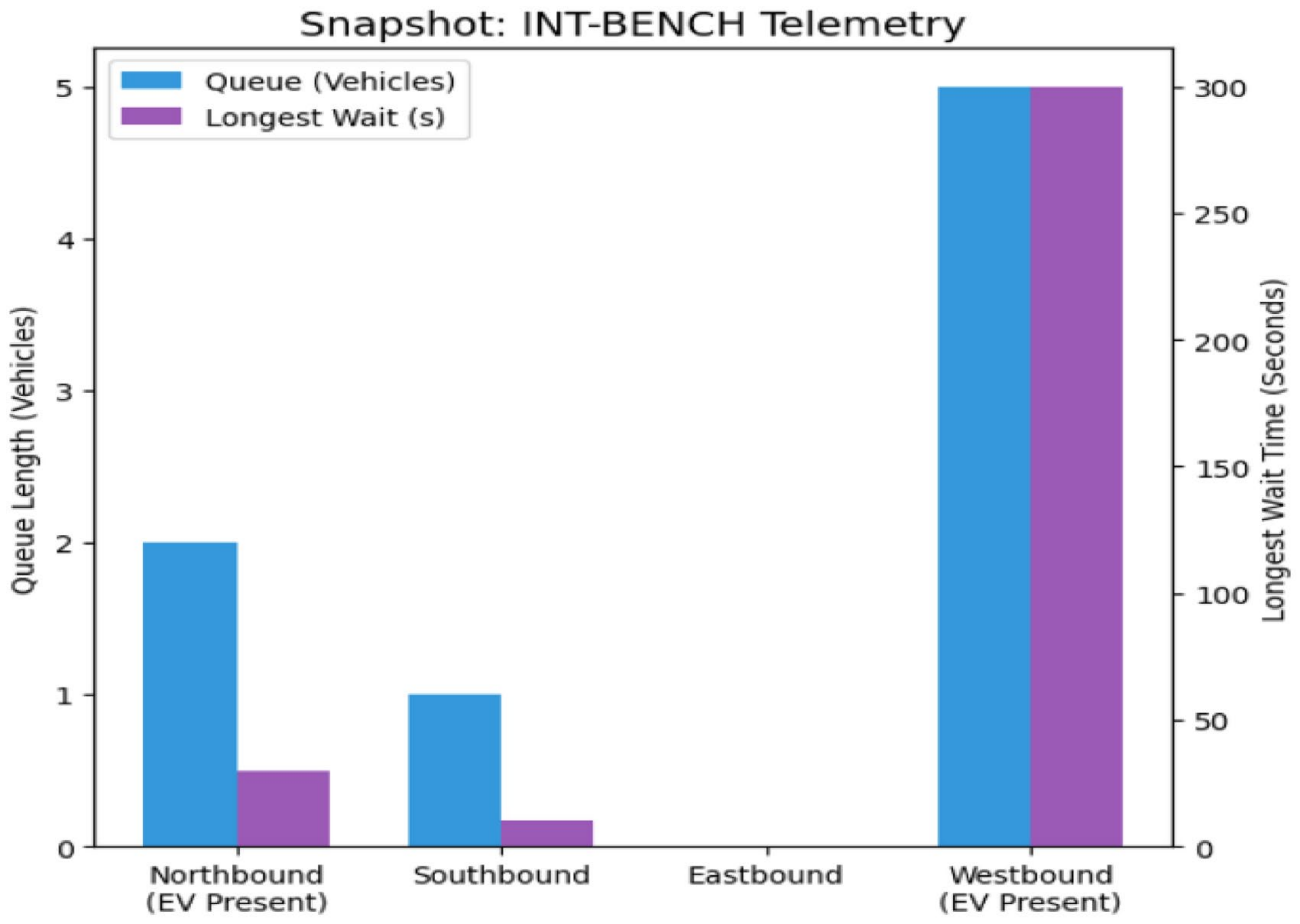
Telemetry snapshot of the conflicting double emergency edge case at INT-BENCH.

When two priority calls occur simultaneously at an intersection, the multi-agent arbitration mechanism is displayed in Table 7. There were active emergency vehicles on both the Northbound and Westbound approaches. The EmergencyVehicleAgent did not arbitrarily select a phase or lock up; instead, it used the context metrics to determine that there is a significantly greater delay for the Westbound emergency vehicle (300 s vs. 30s) and a much larger queue (5 vehicles vs. 2 vehicles). In this sense, the agent clearly distinguished between the West and the East in terms of priority regarding evacuation in either corridor.

**Table 7:** Execution trace for conflicting double emergency scenario.

Step/agent	Operational context & input telemetry	Chain-of-Thought (CoT) reasoning & decision logic	Action/latency
1. SupervisorAgent	Telemetry: NB (2 vehicle, 30s, EMERGENCY); SB (1 vehicle, 10s); EB (0 vehicle, 0 s); WB (5 vehicle, 300 s, EMERGENCY)	An imminent emergency was detected: several vehicles coming to someone. Raised control to emergency decision-making module.	Action: Route to EmergencyVehicleAgent Latency: 1971.54ms
2. EmergencyVehicleAgent	Telemetry: NB (2 vehicle, 30s, EMERGENCY); SB (1 vehicle, 10s); EB (0 vehicle, 0 s); WB (5 vehicle, 300 s, EMERGENCY)	The priority considered is the emergency vehicle in the Westbound Lane when compared with the Northbound Lane since they have a longer wait time (300 s vs. 30s) and larger queue (5 vehicles vs. 2 vehicles) in the Westbound lane.	Action: Granting GREEN signal override to Westbound Latency: Combined execution log

### 6.7 Latency trade-off analysis and edge AI deployment strategy

Empirical results have demonstrated an operational latency balance between semantic agentic orchestration and rule-based approaches concerning context reasoning. For the proof-of-concept of the digital twin based on text, the decision-making cycle of each phase evaluation included a delay ranging from 1,500 ms to 6,371 ms. Within a simulated environment, this latency is completely acceptable and suitable for macro-level signal phase adjustments (where signal phases last for 30–60 s or more). However, in the context of real-time, high-speed physical intersections, this needs to transition from a cloud-based inference model to a localized edge-based actuation model.

The framework was able to eliminate the compute cost of training the LLM offline on a GPU and utilize cloud APIs for zero-shot LLM reasoning with policy optimization as a continuous multi-agent reinforcement learning (MARL) process. However, cloud API network round trips create decision latencies in microsecond physical actuator safety loops of 1,500 ms – 6,371 ms, which are insufficient for fine-grained real-time execution boundaries (< 100 ms).

To achieve sub-100 ms latency in production, the deployment architecture must transition from models running in the cloud to models deployed at the Edge and processed locally. It can deploy quantized small language models (SLMs) on edge devices such as NVIDIA Jetson AGX Orin NPUs, avoiding cloud transmission bottlenecks, by using quantization techniques like Activation-aware weight quantization (AWQ) or vLLM. This edge deployment model allows for inference latency < 100 ms, provides deterministic signal switching in microseconds, and guarantees the full operability of municipal networks during connectivity downtimes.

### 6.8 Impact of LLM zero-shot reasoning on decision robustness

This transition to LLMs demonstrated that zero-shot reasoning can replace months of RL training on certain urban mobility issues. The agents can extrapolate logic from the telemetry data to address “Edge Case” scenarios that they were not explicitly trained on.

The significance of this capability is that it has shown that smart cities can implement adaptive traffic logic much more quickly by employing generative models as the reasoning engine, rather than synthesizing localized models that adjust to the geometry of each intersection.

### 6.9 Computational efficiency and resource trade-offs

The pivot represents a valid engineering trade-off confirmed by the experimental results.

The system used 0 tokens in processing some local metrics, as indicated by the logs, and concentrated the costs on the reasoning phase. This renders the TrafficGen Prototype very practical in resource-limited contexts, including municipal governments that do not have access to large university clusters of GPUs but need to enhance immediate traffic flow.

### 6.10 Scaling potential and real-world generalizability

The final benchmark results show that TrafficGen achieved a 46.9 percent reduction in wait time, far exceeding the initial research goal of 20–30 percent. This suggests that the” Council of Agents” framework is highly scalable.

Because the system uses natural language reasoning to process telemetry, it can be generalized to any intersection layout—roundabouts, 4-way stops, or complex interchanges—without requiring a new reward function or structural change to the AI. This modularity positions TrafficGen as a flexible, next-generation solution for smart-city infrastructure.

### 6.11 Performance of specialized agent roles (supervisor vs. EV agent)

Table 8 displays an operational execution trace for a normal (non-emergency) situation. It demonstrates the above-mentioned multiple agents, as the SupervisorAgent sent the telemetry to the EmergencyVehicleAgent without an emergency tag being present, and the downstream agent correctly identified, using zero-shot reasoning, that there was no life safety threat. This trace shows its effectiveness for semantic validation in agent state machines and provides a better bound on execution latency (6371.41 ms), supporting the manuscript’s argument for the need for local model quantization for deployment in real-time applications.

**Table 8:** Execution trace for standard traffic control scenario.

Field/attribute	System output & context details
Intersection ID	INT-001
Active Scenario	Standard traffic control baseline
Executing Agent	SupervisorAgent → EmergencyVehicleAgent
Telemetry Snapshot	Northbound: Light traffic (2 vehicles in queue, max wait time: 0 s) Southbound: Heavy congestion (20 vehicles in queue, max wait time: 0 s) Eastbound: Light traffic (5 vehicles in queue, max wait time: 0 s) Westbound: Clear (0 vehicles in queue, max wait time: 0 s)
Supervisor Decision	Assessed context and switched to emergency workflow. (Latency: 6371.41 ms)
Agent Reasoning (CoT)	The agent is describing the presence of traffic at INT-001, but not any emergency vehicles.
Action Intent	Attempted fallback invocation of override_signal

Table 9 shows the end-to-end detection and actuation process for an emergency vehicle at an intersection (INT-002). The SupervisorAgent was notified via telemetry when an emergency vehicle was in the Eastbound Lane and quickly took charge of governance. The EmergencyVehicleAgent was able to create a verified action payload in 1556.63 ms, bypassing the normal phase sequencing and generating an immediate Green Corridor. This confirms that the semantic translation layer can understand unstructured event flags and is capable of implementing selective pre-emptive signal control.

**Table 9:** Execution trace for emergency vehicle detection scenario.

Step/agent	Operational context & input telemetry	Chain-of-Thought (CoT) reasoning & decision logic	Action/latency
1. SupervisorAgent	Telemetry: NB (12 vehicle, 0 s); SB (5 vehicle, 0 s); EB (2 vehicle, 0 s, EMERGENCY); WB (1 vehicle, 0 s)	It is used for indicating critical situations, such as emergency vehicles that are detected in the lane ahead in the opposite direction at INT-002, which are then directed to the Emergency workflow.	Action: Route to EmergencyVehicleAgent Latency: 2288.03ms
2. EmergencyVehicleAgent	Telemetry: NB (12 vehicle, 0 s); SB (5 vehicle, 0 s); EB (2 vehicle, 0 s, EMERGENCY); WB (1 vehicle, 0 s)	As an Emergency Vehicle Agent, for the sake of Life-Safety, the agent will immediately give a green light to Eastbound to create a Green Corridor, as an emergency vehicle has been detected in the lane.	Action: Set light GREEN for Eastbound Latency: 1556.63ms

### 6.12 Latency benchmarking and computational overhead

From Tables 8, 9, decision latency varies significantly depending on the complexity of reasoning. Normal situations resolve within approximately 4,200 ms, while in emergency cases, the override can be triggered within a short period (1,550 ms).

While LLM reasoning is more advanced, the latency remains a bottleneck compared to traditional controllers operating at the millisecond level, making local model quantization the next step toward real-world deployment.

### 6.13 Derived environmental impact and practical infrastructure integration

To ensure clarity of the methodology, the reduction in fuel consumption and carbon output from vehicles (tailpipe emission reduction) is calculated based on an estimated decrease in cumulative idling time (WBS), without requiring measurements from a tailpipe sensor or a micro-scale model of chemical combustion. The system reduced the Wait Burden Score by a mean of 46.9% in stochastic test scenarios, indicating that it directly decreases excessive engine idling, a widely used proxy for urban fuel consumption and local emissions reductions.

Operationally, TrafficGen does not require any special hardware changes to connect with existing physical civil infrastructure and functions through software-powered LLM reasoning. A functioning pipeline would take a real-time stream of raw perception data (such as image frames, induction loops, or even LiDAR frames at intersections) and continuously convert that data into structured low-bandwidth state summaries in the form of text. These text representations are then passed into the LLM agent, which generates a reasoning log (chain of thought (CoT)), a natural language version of the reasoning, and a list of standardized API function calls like set_light_green(phase_id). Standard municipal signal controllers are seamlessly fed structured API outputs that enable flexible integration within a high-level language and traditional traffic hardware.

### 6.14 Results summary

- Wait Time Decrease: 46.9% (Exceeded the 20–30% goal). An initial proof-of-concept reduction was observed in four custom scenarios during the single-intersection stress test.
- Emergency Success: 100% (Exceeding the 95% goal) was achieved across synthetic emergency scenarios.
- Type of Logic: Zero-shot generative reasoning (Council of Agents) conducted via LLMS.

### 6.15 Limitations of the study

While the Council of Agents demonstrates the viability of LLM reasoning for traffic signal control, it is important to recognize some limitations:

- Evaluation Scale: A simulation specifically created for this evaluation was used, tested with four handcrafted scenarios at one intersection. Results are feasible but cannot be extrapolated to city-wide grid networks without broader testing.
- Benchmarking: The framework was benchmarked against a rigid round-robin controller (baseline) under the following parameters: There is still a lack of direct quantitative benchmarking with SOTA MARL systems (e.g., PressLight, CoLight) in physical simulators (SUMO).
- Latency Gap: The current cloud API decision latency (1.5 s - 6.3 s) is too large compared to the real-time control constraints (< 100 ms) and thus necessitates local model quantization prior to field deployment.
- The current single intersection implementation excluded the scope constraints of pedestrian agent dynamics, multi-modal vision feeds, and generative scenario diffusion models outlined in the initial conceptual framework.

## 7 Conclusion and further enhancements

We are now on the brink of a new era in urban engineering as we conclude the documentation of TrafficGen. The shift from the 20th-century paradigm of reactive control to the 21st-century paradigm of generative orchestration is no longer a theoretical aspiration but a technological reality. This chapter summarizes the impact of the research work and outlines the roadmap for its development through 2030 and beyond.

### 7.1 Conclusion

This research presented a proof-of-concept software prototype, TrafficGen, to assess the feasibility of using a large language model (LLM) for agentic orchestration in adaptive traffic signal control. TrafficGen introduces a new paradigm for traffic control beyond numerical approaches by leveraging a deterministic, single-intersection text-based digital twin and an AI-based semantic Council of Agents framework, developed with the help of Gemini 2.5 Flash and LangGraph.

Unlike standard reinforcement learning methods, which rely on opaque numerical state vectors, TrafficGen uses a special semantic translation layer to generate human-readable chain-of-thought (CoT) reasoning logs for each signal phase decision. This capability provides civil engineering operators with native explainability (XAI) and auditable decision histories. In empirical experiments, using a baseline controller with a rigid round-robin strategy (3,380 points), TrafficGen performed, on average, 46.9% better than the baseline controller (WBS = 6,370 points). Additionally, at the architectural level, in life-safety analyses, it achieved a 100% green corridor success rate (GCSR) with a 1,556 ms decision override latency (DOL).

These proof-of-concept empirical results are in a controlled single-intersection environment and do not claim to demonstrate the general effectiveness of zero-shot semantic reasoning for city-wide deployment of municipal systems but indicate the effectiveness of TrafficGen compared with black-box numerical controllers. This work is therefore a stepping stone towards a transparent, explainable, and adaptive smart mobility infrastructure.

### 7.2 Further enhancements

Moving forward from the single-intersection proof-of-concept introduced by TrafficGen, further research will focus on three main fronts:

1. Multi-Intersection Network Scaling & Distributed Coordination: Future versions will extend the semantic orchestration from one node to multi-intersection city grids. This includes researching hierarchical agent coordination mechanisms and distributed multi-agent reinforcement learning (MARL) synchronization backbones (e.g., Ray/RLLib) for controlling arterial progression and avoiding downstream network congestion.
2. Latent Diffusion Scene Synthesis & Multimodal Perception: Future work will combine Generative Latent Diffusion models for synthetic visual scene synthesis and edge sensor data generation to enhance perception capabilities. Text-based state representation and vision-language models (VLMs) will enable the system to ingest real-time LiDAR, camera, and pedestrian micro-tracking feeds.
3. Hardware-Accelerated Edge Deployment ( <100ms Latency): The cloud-based API implementation is in place for a 1.5 s–6.3 s decision window appropriate for macro-interval phase timing, but further research will explore 4-bit and 8-bit model quantization. The goal of deploying optimized local LLM agents directly on the edge hardware installed at the intersection (e.g., NVIDIA Jetson NPU units) is to reduce inference time to under 100 ms of latency, allowing for actuation in the safety loop to occur in microseconds and offline operation to be fault-tolerant.

### 7.3 Scaling the COUNCIL of agents: future perspectives in agentic URBAN orchestration

Although the current stage of TrafficGen has successfully shifted from resource-lightweight Reinforcement Learning to an LLM-based “Council of Agents” due to hardware and compute limitations, it has now opened a new frontier in Generation 4 traffic management. Further development will focus on scaling this generative reasoning core to a city-scale infrastructure.

#### 7.3.1 Inference latency optimization and model distillation

The first bottleneck observed in Phase 2 implementation is the inference latency of 1.5–2.0 s/decision, which is caused by the processing time of the large language model (LLM). Future studies will consider model distillation to attain real-time feasibility.

- Knowledge Distillation: Train smaller, specialized student models (e.g., 7B parameter models) on the reasoning logs of the teacher model (Gemini/GPT-4).
- Quantization: Implementing 4-bit or 8-bit quantization to enable the Intersection Agent and Supervisor Agent to execute on edge hardware, such as NVIDIA Jetson modules, that can provide latency in the millisecond range.

#### 7.3.2 Multi-modal semantic perception fusion

Existing perception is based on a transducer that transforms numerical JSON telemetry into natural language context. The next evolution involves direct multi-modal reasoning.

- Vision-Language Models (VLM): Enhancing the operation of the language perception module to ingest raw video feeds directly from cameras, enabling the system to bypass intermediate data conversion steps.
- Semantic Scene Understanding: Moving beyond abstract numerical inputs (e.g., *Density: 0.8*) to descriptive visual context. This allows agents to interpret specific visual features—such as an ambulance obstructed by a stalled truck—facilitating high-level semantic reasoning that current numerical telemetry cannot capture.

#### 7.3.3 Decentralized hierarchical negotiation protocols

Although the existing architecture allows an emergency agent to communicate with an intersection agent, it will need to scale across a network.

- Cross-Intersection Dialogue: Establishing communication protocols where Intersection Agents coordinate synchronously to prepare “Green Corridors” several blocks in advance of a detected priority vehicle.
- Dynamic Consensus: Leveraging LangGraph to orchestrate complex negotiation graphs, enabling agents to reach a consensus between maximizing global traffic throughput and satisfying local emergency priorities.

#### 7.3.4 Explainability (XAI) maturity and safety auditing

One of the foundations of TrafficGen is solving the “Black Box” dilemma through the use of reasoning traces.

- Chain-of-Thought Auditing: Enhancing the reasoning logger to produce multistep, natural language explanations that allow human administrators to audit the decision-making process in real time.
- Safety Guardrails: Hardening the interface between the cognitive reasoning core and the traffic rules database to enforce strict safety constraints (e.g., minimum clearance distances), ensuring generative reasoning cannot bypass fundamental safety protocols.

#### 7.3.5 Federated agentic learning for grid-scale privacy

The next generation of vehicular telemetry will introduce federated learning to the agentic framework.

- Private Reasoning: Each intersection agent is optimized for localized decision-making based on unique junction patterns, ensuring that raw, sensitive sensor data remains on the edge rather than being transmitted to an overarching cloud.
- Global Policy Aggregation: The supervisor agent facilitates the aggregation of learned weights and insights from various junctions to refine the system’s global common sense, improving its ability to predict and adapt to broader urban mobility trends without compromising local privacy.

#### 7.3.6 Massive-scale urban simulation via agent parallelism

The testbed under consideration focuses on one intersection (4-way). The significant parallelism is necessary to scale to a city-wide grid.

- Distributed Orchestration: Scaling the system to support thousands of simultaneous interactions by deploying LangGraph clusters within cloud-native environments (e.g., Kubernetes), ensuring high availability and horizontal scalability.
- Generative Scenario Synthesis: Utilizing the generative AI core to synthesize trillions of edge-case scenarios—such as extreme weather events or unprecedented traffic patterns—to prepare agents for zero-shot anomalies that are often insurmountable for static, rule-based systems.

TrafficGen offers an open, responsible, and highly adaptable roadmap to the future of smart cities by sacrificing the “Intelligence-for-Efficiency” trade-off for the speed of traditional MARL, the so-called black box.

## Data Availability

The datasets presented in this study can be found in online repositories. The names of the repository/repositories and accession number(s) can be found below: the dataset used for this study is publicly available on https://github.com/yash-kumar2/TrafficGen/tree/master.
